# Current and Future Impacts of Lithium Carbonate from Brines: A Global Regionalized Life Cycle Assessment Model

**DOI:** 10.1021/acs.est.4c12619

**Published:** 2025-03-26

**Authors:** Vanessa Schenker, Stephan Pfister

**Affiliations:** Chair of Ecological Systems Design, Swiss Federal Institute of Technology Zurich, Laura-Hezner-Weg 7, CH-8093 Zurich, Switzerland

**Keywords:** metals, life cycle inventory, salars, geothermal brines, batteries, mining

## Abstract

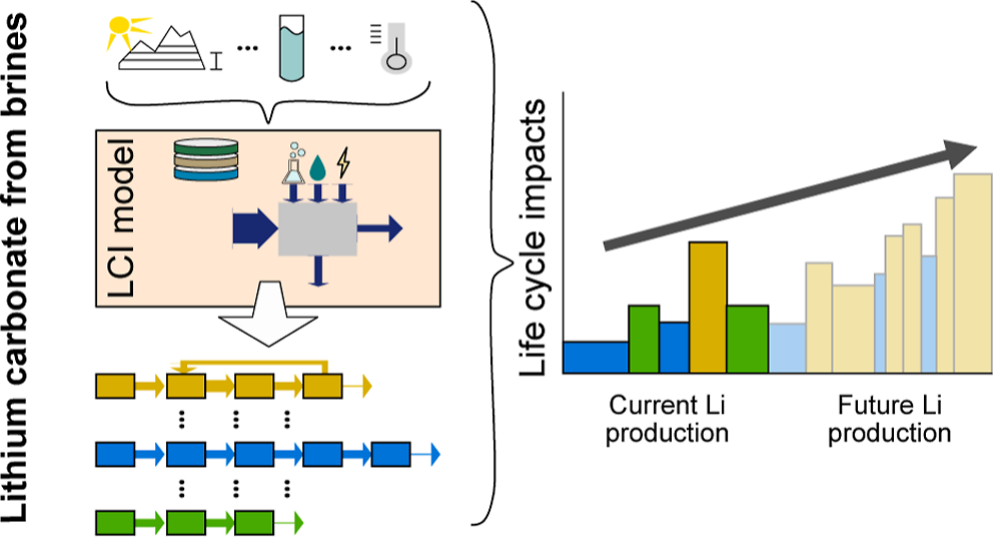

Lithium (Li) is essential for decarbonization strategies, such as electric vehicles and renewable energy storage, which experiences the largest growth rates among metals required for low-carbon technologies. To meet this demand, the raw materials sector must increase current capacities and develop new capacities at untapped deposits. Understanding life cycle impacts is crucial to avoid severe environmental burden shifts in the future. Although site-specific life cycle inventories exist, they do not allow for a comprehensive global assessment of the Li sector, particularly in capturing technological developments. To address this, our study presents a life cycle inventory model for brines that maintains essential site-specific parameters while providing a global perspective. We define core parameters for site-specific modeling of Li carbonate (Li_2_CO_3_) production and develop a systematic approach to addressing data gaps. Our model employs a class-based structure for 30 mapped processes from the literature and quantifies environmental and technical flows. Overall, we cover 25 sites, representing 300 kilotonnes (90%) of current Li_2_CO_3_ production from brines and an additional 315 kilotonnes of potential future production. One key finding is that sites using direct Li extraction have 7-fold higher climate change impacts than sites using conventional technologies on average, while water scarcity impacts are doubled on average. The difference is a result of the larger brine mass required to be treated due to lower Li grades. Furthermore, our model allows the implications for Li-ion battery production to be analyzed.

## Abstract 2

Life cycle impacts of lithium carbonate from brines are underestimated in the literature. Our global, regionalized life cycle inventory model demonstrates increasing impacts due to technology choices and lower brine quality in the future.

## Introduction

The demand for lithium (Li), driven by its critical role in energy storage systems, has intensified the focus on its extraction and processing.^1^ Li is the metal seeing the highest growth rates of metals required for low-carbon technologies.^2,3^ Worldwide capacities to extract Li-bearing brines and minerals need to increase, and new sites need to be developed.^4−6^ This growth presents challenges, as increasing production at existing sites and opening sites will impose additional environmental burdens. However, these are difficult to quantify due to the low data availability of the mining sector.^7,8^

Li mining is nowadays concentrated in Australia, Chile, Argentina, and China,^9^ which have significant deposits of Li-bearing brines and minerals. In 2022, 43% of Li chemicals, measured in Li carbonate equivalent (LCE), were derived from continental brines located in salt lakes/salars, while the remaining 57% came from minerals.^10^ Meanwhile, exploration activities have intensified globally. The “Li triangle” in Argentina, Bolivia, and Chile has remained a focal point due to its Li-rich brines in salars.^10−12^ This region is expected to be one of the main future Li suppliers.^13,14^ Beside this, there is increasing interest in other Li-bearing deposit types, such as geothermal and oilfield brines located in North America and Europe.^4,10,15^

The common products are Li carbonate (Li_2_CO_3_) and Li hydroxide monohydrate (LiOH·H_2_O).^16,17^ Various technologies exist to extract Li from brine and produce these chemicals.^18^ Technological choices in Li_2_CO_3_ production from brines are influenced by site-specific conditions, particularly the unique brine chemistry.^15,19^ Both Li concentration and the presence of other ions (e.g., Mg, B, Fe, and Si), which can complicate processing, influence the applied technology.^16^ Furthermore, environmental conditions (e.g., the rainy season in Bolivia complicates the use of evaporation ponds) also play a crucial role.^20^ Hence, the resource demand for producing Li chemicals, and thus the associated environmental impacts, can vary widely.

Research in life cycle assessment (LCA) of Li chemical production is focused on specific sites characterized by unique geological and technological conditions.^21−27^ These site-specific studies provide valuable insights into individual environmental impacts. Although Ambrose and Kendall^28^ present life cycle impacts of Li chemicals on a global scale, their study lacks the granularity needed to accurately assess the diverse processing technologies and to discuss improvements from a life cycle perspective. Schenker et al.^22^ present systematic guidelines to perform LCAs of Li_2_CO_3_ from brines and to allow comparability between sites. However, their framework is applied to only five existing and future sites. The sensitivity of process-related parameters (e.g., process temperatures, adsorption capacity, etc.) is not assessed, prohibiting any evaluation of life cycle impact drivers and prioritization of data collection. Recognizing the uncertainties related to input data, Schenker et al.^27^ address future Li_2_CO_3_ production from two geothermal sites by assessing a large number of scenarios with systematic variations of brine chemistry and drilling activity as the main focus of their study. However, the small number of assessed sites in these two studies does not (1) allow us to understand the relation between brine chemistry, applied technology, and life cycle impacts and (2) cover enough sites to generalize these findings for the current and future Li_2_CO_3_ market. Consequently, the development of a systematic LCA model that encompasses a larger spectrum of processing technologies, treats the mosaic pattern of site-specific data, and addresses the inherent uncertainties is crucial for accurately evaluating the environmental impacts on a global scale.

This study introduces a novel global LCA model of existing and future Li_2_CO_3_ production from brine deposits, aiming to bridge the identified research gaps. Our model not only facilitates a broader understanding of the life cycle impacts of the current Li production but also enables the exploration of potential environmental impacts associated with future expansion of Li mining activities. By employing a parameterized and modular approach, the model accommodates a wide range of production technologies and site conditions. In total, life cycle inventories (LCI) of 25 sites across Argentina, Bolivia, Chile, China, the United States, and Germany are modeled and discussed. LCI are used to systematically assess climate change and water scarcity impacts. Additionally, we present results on reduction potentials of climate change impacts by integrating renewable energy provision. Given the critical role of Li in the Li-ion battery (LIB) sector, we discuss the implications for LIB manufacturing. As a last point, the robustness of the model is assessed in detail.

## Li_2_CO_3_ Production from Brines

**Figure 1:**
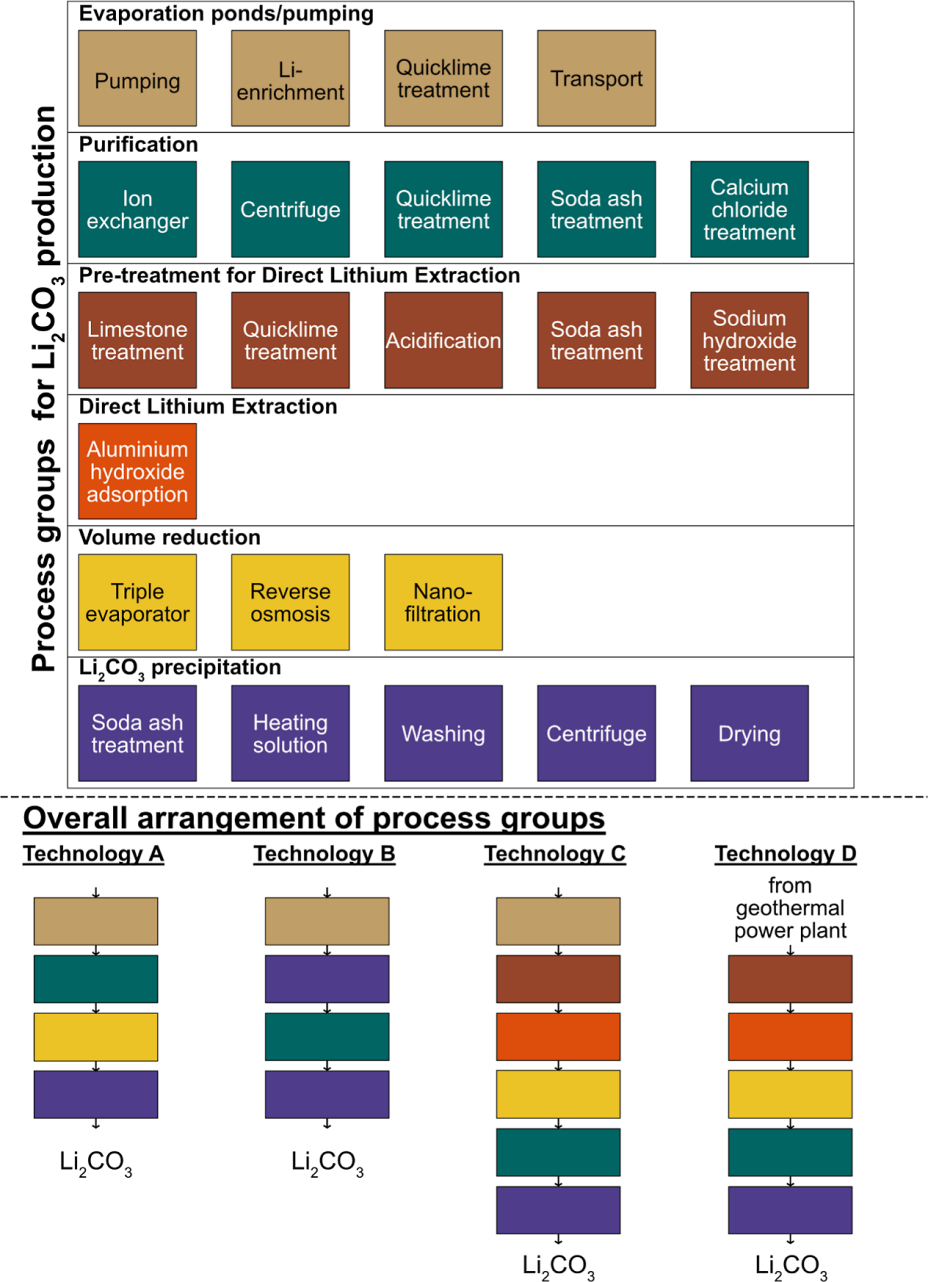
Overview of reported processes for Li_2_CO_3_ production from brines. Each process is grouped according to its primary function within a lithium brine processing pathway. The bottom panel illustrates the four defined technology groups in a generalized manner, as the selection and arrangement of the processes are site-specific. Site-specific flow sheets can be found in Table B.12. (A) Conventional chemical-based technology, (B) conventional ion-exchanger technology, (C) direct Li extraction (DLE) technology, and (D) DLE technology from geothermal brines.

Processing pathways, adapted to site-specific conditions,^15,16,29,30^ follow distinct patterns (Figure 1). Currently producing sites, such as Atacama, are characterized by the use of evaporation ponds to increase the Li concentration using solar energy.^19^ Common practice is to add chemicals, such as quicklime (CaO), to remove impurities (e.g., Cauchari-Olaroz and Pastos Grandes). Once the brine reaches a site-specific Li concentration, it undergoes further purification with organic solvents, soda ash (Na_2_CO_3_), sodium hydroxide (NaOH), and/or calcium chloride (CaCl_2_). Technical grade Li_2_CO_3_ is produced by adding Na_2_CO_3_, and if the battery grade is desired, this is dissolved in water at low temperatures and reheated to precipitate higher purity Li_2_CO_3_ (battery grade). This study refers to this processing pathway as conventional chemical-based technology (Type A).

While Type A mainly relies on chemicals for purification, another pathway uses ion exchangers to remove impurities, as seen at Silver Peak and Olaroz. They use evaporation ponds and CaO to concentrate the brine in a first step similar to Type A but then directly precipitate impure Li_2_CO_3_ with Na_2_CO_3_ (Figure 1). The low-grade Li_2_CO_3_ is then dissolved in water at low temperatures, and the LiCl solution is passed through multiple ion exchangers to remove impurities, such as Mg, Ca, and B. The purified brine is reheated to precipitate battery-grade Li_2_CO_3_.^31,32^ Our study group sites use this technology into the group conventional ion-exchanger technology (Type B).

DLE is used for salar-related brines (e.g., Hombre Muerto and Chaerhan) and is being considered for geothermal brines (e.g., Salton Sea and Upper Rhine Graben).^33^ DLE technologies include ion-exchange resins, liquid–liquid extraction, nanofiltration, and electromembranes.^15,34^ DLE technologies allow access to deposits with lower Li concentrations and/or, in general, unfavorable brine chemistry. However, data on DLE are often unavailable.^33^ The general DLE pathway involves pretreatment depending on brine chemistry (e.g., ion exchange, precipitation, solvent extraction, and acidification), chosen DLE technology, and postprocessing (further refining and volume reduction).^35^ For example, geothermal brines with high Fe and Si concentrations require specific precipitation reactions to pretreat the brine, while continental brines need Mg and Ca removal.^15,36^ Postprocessing includes ion exchangers, and precipitation and volume reduction to precipitate Li_2_CO_3_, with the byproduct freshwater, which is used for other processes.^35^ When the brine is purified, technical Li_2_CO_3_ is precipitated, and the purity can be increased by the aforementioned processes. Our study refers to sites using DLE for continental brines as DLE technology (Type C) and for geothermal brines as DLE technology (Type D) due to the different required pre- and post-treatments.

## Methods

This study conducts a comprehensive LCA of Li_2_CO_3_ production from brines on a global scale, adhering to the ISO 14040 and 14044 standards for LCA.^37,38^ The goal is the development of a parameterized and modular model to quantify LCI of existing and future Li_2_CO_3_ production from brines (the model is accessible via the github link), with a global scope. The functional unit is 1 kg of Li_2_CO_3_ at the battery grade. The scope encompasses all stages from brine extraction to final Li_2_CO_3_ production. Life cycle impacts of 1 kW h capacity of a LIB cell with an NMC cathode (NMC811 = nickel: 80%, manganese: 10%, and cobalt: 10% by mass) and one with an LFP cathode composed of Li, Fe, and PO_4_ from ecoinvent^39^ are assessed when using Li_2_CO_3_ LCI from this study.

### LCI Modeling

We utilize the database from S&P Global,^10^ selecting active mine sites and exploration sites, particularly those with published feasibility or technical reports. We assess 25 sites in Chile, Argentina, Bolivia, China, the United States, and Germany. We integrate the reported processing sequence and input data of 5 salar-related sites (Atacama, Olaroz, Cauchari-Olaroz, Hombre Muerto (North), and Chaerhan)^22^ and 2 geothermal sites (Salton Sea, Upper Rhine Graben)^27^ in our study.

### Input Data and Treatment of Data Gaps

The selection of required parameters is based on Schenker et al.^22^ However, these data are highly scattered and often not reported, prohibiting sound assessments on a global level. Input data of all assessed sites and their references can be found in Table B.2. Recognizing these challenges, we present a structured, hierarchical approach to data collection.

At the core of our model are critical data, such as geographic location (country, elevation, longitude, and latitude) and site characteristics (deposit type, salar or geothermal, brine chemistry, and technology). Based on the geographic location, the model uses a global data set on monthly recorded temperature from the year 2019 to obtain the annual air temperature of the specific location by using the geographically closest data point.^40^ If the evaporation rate is not reported in the literature, the model uses the value of the closest site that reported the evaporation rate in the provided database. The elevation is used as a proxy to determine the boiling point on site, a necessity to quantify the heating demand of specific processes. The inconsistent reporting of the brine’s elemental composition presents another layer of complexity. The brine chemistry is obtained from scientific literature, patents, and technical reports.^12,19,24,31,41−55^ If brine chemistry is only partially reported, the model approximates the brine chemistry by using that from the closest salt lake in our database. Detailed information on the technology used at each site is essential, ideally at a process level.^15,22,23,32,42−50,52−54,56−74^ If such detailed information is not available and only high-level descriptions of the technology are provided, the model defaults to a predefined set of processes that approximates the described technology. For example, sites often report the use of DLE technology,^48^ while the site-specific purification steps are not published. Hence, the model approximates purification steps (Mg removal by soda ash and acidification) following an direct lithium extraction sequence, as described in Vera et al.^33^ and Mousavinezhad et al.^75^ Other operational data (e.g., operating days, lifetime, well depth, etc.) required to model the LCI are used when they are specifically reported and if not, then standard values defined in the model are used.

### Site-Specific Li_2_CO_3_ Production

Our model employs a class-based structure for each process, allowing for dynamic execution (Figure A2). In total, 30 processes are included. The model enables the calculation of environmental and technical flows, including water and energy demand, chemical usage, and waste generation, tailored to the site-specific conditions. Our model checks mapped dependencies between processes to prevent illogical processing sequences. Processes are modeled based on the incoming mass flow from either a previous process or the initial brine mass flow used to produce a defined amount of Li_2_CO_3_. Each process contains a predefined set of technical and environmental flows. The iterative approach to model LCI based on patents, technical reports, and literature by Schenker et al.^22^ is used to systematically model process-related flows. This is further expanded by parameterizing 68 process-related parameters (e.g., process temperatures and pulp concentrations) and defining ranges to account for uncertainties related to the modeling approach.

The heat demand for specific processes is calculated based on thermodynamics by using the process-specific temperature and the temperature of the incoming pulp. The heat capacity of the incoming solution as well as latent heat due to the phase changes is used to calculate the heat demand. In addition, a heat loss of 15% is added to the modeled demand.^76^ Heat recovery is integrated in multiple processes when water and/or pulp flows are recirculated (e.g., water generated in a mechanical evaporator and reverse osmosis).

Any demand for chemicals is modeled using stoichiometric reactions. Chemicals are added to force a specific precipitation of wanted or unwanted ions, to wash pipes, or to adjust the pH of the brine. Chemicals, such as organic solvents or cationic resin, can be used multiple times, which is taken into account by including reported recycling rates from the literature. Waste in these processes is generated by either centrifuges (as a subsequent process) or sedimentation in the presented model. Whether the waste is a liquid or solid is determined by the process configuration.

Water is required to dilute chemicals, wash Li_2_CO_3_, desorb Li from the resin, and dissolve Li_2_CO_3_. For chemicals, the water demand is determined by the mass flows of chemicals and a defined surplus to account for incomplete chemical reactions. Water used for washing activities is defined based on the incoming mass flow.

Electricity demand is calculated using literature values per incoming mass flow of the specific process.

### Site-Specific Databases and Regionalization of the Background Database

In a next step, the model creates site-specific databases in Brightway 2.0.^77^ Required flows from ecoinvent v3.9.1 are mapped in the model and can be extended. The model chooses country- or region-specific data sets existing in ecoinvent v3.9.1.^39^ To assess water scarcity impacts, biosphere flows, such as freshwater losses due to evaporation when liquid waste is stored, are regionalized.

### Life Cycle Impact Assessment

Assessed life cycle impacts are based on the literature highlighting their relevance regarding Li mining.^21,22,24,26^ We assess climate change impacts utilizing the IPCC method assessing the global warming potential of emissions over a 100 year time horizon (GWP100a)^78^ and regionalized water scarcity impacts following the AWARE methodology.^79^ Site-specific characterization factors of AWARE are documented in the Supporting Information. As highlighted by Halkes et al.,^80^ some salars have spatial overlaps with multiple characterization factors due to the low watershed resolution of the WaterGAP model, while for other salars, no characterization factor is available at all. Four operations are located in areas with no characterization factors. We fill these gaps by using updated AWARE characterization factors provided by Pfister and Scherer.^81^ There has been a discussion on the integration of brine consumption in the AWARE methodology.^25,80^ Studies by Kelly et al.,^24^ Chordia et al.,^21^ and Schenker et al.^22^ do not include the water content of the brine in their assessments when assessing water scarcity. As AWARE is designed to only assess freshwater consumption, we follow the approach defined by the UNEP-SETAC Life Cycle Initiative.^79^

### Local Sensitivity Analysis

The robustness of our model is tested by performing a local sensitivity analysis (LSA) for each site. In total, we vary 68 parameters relevant for the 30 mapped processes in our model. The ranges are systematically defined based on the parameter’s origin (for rule definition, see the Supporting Information, “Local Sensitivity Analysis”). Patent-derived parameters are varied by the reported ranges within the patents. If parameters from scientific literature or technical reports are obtained from sources reporting ranges (e.g., adsorption capacity in Vera et al.^33^), we use these values. If there are no ranges reported, we define the ranges based on systematic rules (Table B.11). If there are parameters derived from proxies due to the lack of literature, we increase the defined value range accordingly. For each iteration, we quantify climate change and water scarcity impacts of 1 kg of Li_2_CO_3_ at the battery grade. In addition to that, we use brine chemistry data^55^ to test the sensitivity of the results (Table B.3).

## Results and Discussion

### Life Cycle Inventories

**Figure 2:**
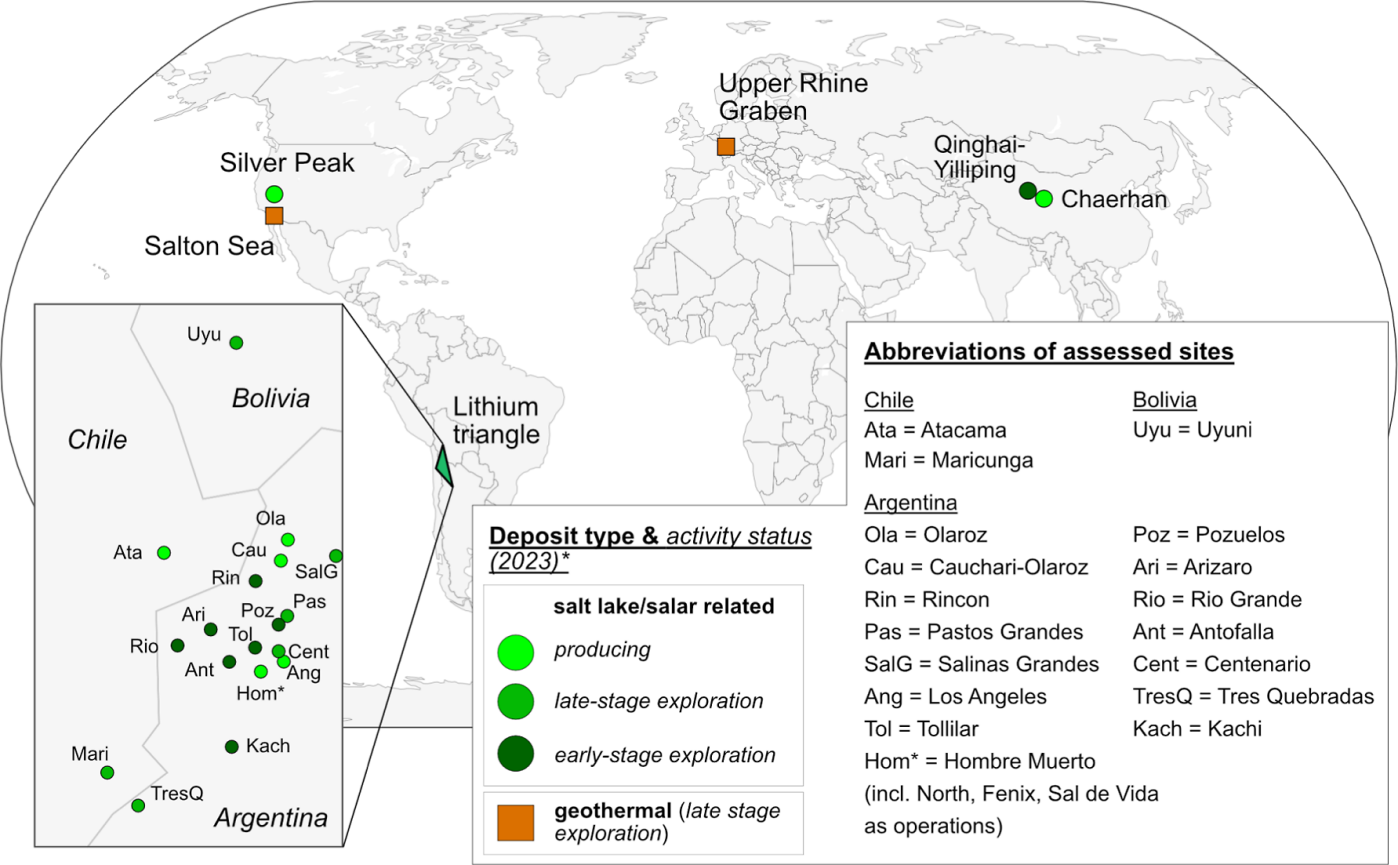
Global map of assessed Li sites from either salt lakes/salars or geothermal brines. Data (Table B.2) are taken from S&P Global.^10^

This section describes the modeled LCI of 25 sites based on the availability of technical reports, patents, and literature (Figure 2). We cover 300 kilotonnes (kt) of current LCE production representing 90% of the LCE production in 2022^10^ and additional 315 kt of future LCE production from brines. Notably, the capacity of future production in this study is based on announcements and, thus, faces high uncertainties. Missing production capacities of 8 exploration sites were filled by assuming a midsize operation (10 000 t Li_2_CO_3_/year). The sites are divided into four defined technology groups. Required input data and LCI of each site can be found in the Supporting Information (Tables B.2 and B.11).

### Conventional Chemical-Based Technology—Type A

Seven of the 25 sites reported a chemical-based procedure to produce Li chemicals. The brine’s Li concentrations vary between 0.04 and 0.15 wt % Li with Atacama having the highest Li concentration (Table 1). Heat demand varies between 13 and 49 MJ/kg of Li_2_CO_3_, and power demand varies between 1.1 and 2.5 kW h/kg of Li_2_CO_3_. The freshwater demand ranges between 0.04 and 0.2 m^3^/kg of Li_2_CO_3_. The chemical demand (i.e., CaO and CaCl_2_) is highly variable as it is determined by the brine chemistry (Table 1) and purification processes. For example, Pastos Grandes has the highest CaO demand, with 6.2 kg/kg Li_2_CO_3_, while the lowest is reported to be 0.02 kg/kg Li_2_CO_3_ at Atacama. The brine from Pastos Grandes has the lowest Li concentration and a high impurity concentration compared to the other brines leading to this elevated CaO use. Na_2_CO_3_ use is similar across the sites due to the main demand coming from the Li_2_CO_3_ precipitation, which is not affected by site-specific conditions.

**Table 1:** Material and Energy Demand Per kg of Li_2_CO_3_ (Battery Grade)^a^

site	technology	Li (wt %)	impurity (wt %)	heat (MJ)	power (kW h)	process water (kg)	CaO (kg)	H_2_SO_4_ (kg)	Na_2_CO_3_ (kg)	HCl (kg)	NaOH (kg)	resin (kg)	other (kg)	waste (kg)
Atacama	A	0.15	2.7	18	1.1	47	0.03	0.03	2.0	0.06	—	0.03	0.06	112
Tres Quebradas	A	0.10	4.4	24	1.7	53	—	0.04	1.9	0.11	—	0.06	0.12	174
Maricunga	A	0.09	1.8	49	1.6	200	0.2	0.13	2.5	0.13	—	0.06	0.81	132
Sal de Vida	A	0.08	1.1	13	1.1	33	0.4	0.00	1.9	0.00	—	0.00	0.00	27
Hombre Muerto North	A	0.07	1.1	48	1.3	61	3.4	0.05	1.9	0.28	—	0.08	0.69	261
Cauchari-Olaroz	A	0.05	0.9	24	1.2	56	2.7	0.00	1.9	0.19	—	0.08	0.36	226
Pastos Grandes	A	0.04	0.3	18	2.5	85	6.2	0.08	1.9	0.16	—	0.09	0.20	167
Olaroz	B	0.06	1.8	9	1.2	128	3.7	0.11	1.9	0.03	—	0.02	—	113
Silver Peak	B	0.02	0.7	8	1.4	67	0.3	0.00	1.9	0.03	—	0.02	—	93
Uyuni	C	0.07	3.5	241	3.9	336	—	0.04	37.6	3.96	0.5	0.04	—	101
Fenix	C	0.07	1.1	352	4.2	247	—	0.08	9.7	2.51	0.4	0.04	—	124
Rio Grande	C	0.03	1.3	388	4.2	261	—	0.08	13.0	6.77	0.5	0.04	—	124
Centenario	C	0.03	1.2	387	4.3	265	—	0.09	12.7	4.05	0.5	0.04	—	130
Rincon	C	0.03	1.6	430	4.4	269	—	0.10	12.7	8.88	0.5	0.04	—	134
Tolillar	C	0.03	0.2	442	5.4	269	—	0.11	11.3	3.74	0.5	0.04	—	139
Arizaro	C	0.03	3.1	457	4.5	324	—	0.11	25.0	19.55	0.5	0.04	—	139
Antofalla	C	0.03	0.8	446	4.5	252	—	0.11	6.6	5.42	0.5	0.04	—	141
Salinas Grandes	C	0.02	0.4	524	4.6	272	—	0.13	9.4	3.14	0.5	0.04	—	150
Kachi	C	0.02	*	546	6.1	304	—	0.14	15.5	4.31	0.5	0.04	—	158
Los Angeles	C	0.02	1.0	561	4.8	281	—	0.15	9.0	7.36	0.5	0.04	—	161
Pozuelos	C	0.02	1.1	656	5.0	328	—	0.17	17.9	11.29	0.5	0.04	—	172
Chaerhan	C	0.02	*	465	10.0	212	—	0.13	1.9	0.19	0.5	0.09	—	80
Qinghai Yiliping	C	0.02	*	444	10.0	212	—	0.13	1.9	0.19	0.5	0.09	—	80
Upper Rhine Graben	D	0.02	0.5	179	14.0	356	0.05	—	1.9	1.46	1.0	0.28	0.38	23
Salton Sea	D	0.02	2.6	172	13.8	357	2.4	—	1.9	2.05	1.0	0.28	3.72	37

a Impurity concentration signed with an * means that there was no information on the impurity concentration and a proxy was used. The column “other” presents the sum of other chemicals used on-site. (A) Conventional chemical-based technology, (B) conventional ion exchanger technology, (C) DLE technology from continental brines, and (D) DLE technology from geothermal brines.

### Conventional Ion Exchanger Technology—Type B

Olaroz and Silver Peak produced Li_2_CO_3_ using ion exchangers to purify the brine. The Li concentration is 0.02 wt % Li at Silver Peak and 0.06 wt % Li at Olaroz.

The heating demand is 8 MJ/kg of Li_2_CO_3_ at Silver Peak and 9 MJ/kg of Li_2_CO_3_ at Olaroz. Power consumption is 1.2 kW h/kg Li_2_CO_3_ at Silver Peak and 1.4 kW h/kg Li_2_CO_3_ at Olaroz. Water demand is higher compared to Type A, with 0.07 m^3^/kg of Li_2_CO_3_ at Silver Peak and 0.13 m^3^/kg of Li_2_CO_3_ at Olaroz due to water use during the regeneration of ion exchangers. CaO consumption is estimated at 0.36 kg/kg of Li_2_CO_3_ at Silver Peak and 3.7 kg/kg of Li_2_CO_3_ at Olaroz. The discrepancy is explained by the smaller mass of Mg that needs to be removed in the brine of Silver Peak. Additionally, 1.9 kg of Na_2_CO_3_/kg of Li_2_CO_3_ is required at both sites to precipitate impure Li_2_CO_3_.

### DLE Technology—Type C

Fourteen sites report the installation of DLE technology. Most assessed sites are located in Argentina, with Uyuni in Bolivia and Chaerhan and Yiliping in China as exceptions. The Li concentration varies between 0.02 and 0.07 wt % Li, with a notable tendency to lower Li grades than in Type A and B. The heat demand ranges between 241 and 656 MJ/kg Li_2_CO_3_, which is much more than the Types A and B. The highest energy demand is modeled for the operation at Pozuelos and results from the relatively low Li grade compared to the other sites. Power demand ranges between 3.9 and 10 kWh/kg Li_2_CO_3_, while water demand ranges between 0.21 and 0.34 m^3^/kg Li_2_CO_3_. Water and electricity demand are also much higher than those for Type A and B. Na_2_CO_3_ demand varies between 1.9 and 38 kg/kg Li_2_CO_3_, with Uyuni having the highest Na_2_CO_3_ demand. This is a result of the elevated Mg concentration in the brine compared to other brine sites and requires removal by Na_2_CO_3_. The lowest hydrochloric acid (HCl) demand for acidification is modeled for Fenix with 2.5 kg/kg of Li_2_CO_3_. The highest demand is modeled for Arizaro with 19 kg/kg Li_2_CO_3_ due to the elevated impurity (especially SO_4_) concentration of the brine compared to sites such as Fenix.

### DLE Technology—Type D

DLE will be used for Li extraction from geothermal brines at the Salton Sea in the United States and Upper Rhine Graben in Germany. The Li concentration in the geothermal brine is 0.19 wt % at Upper Rhine Graben and 0.18 wt % at Salton Sea. As aforementioned, processing sequence and input data are primarily sourced from Schenker et al.^27^ and integrated into the model to extend it to potential future geothermal sites. The energy requirements are 172 MJ/kg Li_2_CO_3_ at Salton Sea and 179 MJ/kg Li_2_CO_3_ at Upper Rhine Graben. Power demand is similar for both sites, at 14 kW h/kg Li_2_CO_3_, as is water demand, at 0.36 m^3^/kg Li_2_CO_3_. While heat demand is lower than for Type C sites, water consumption and electricity demand are higher due to more pumping of brine/process water and the lower adsorbent capacity reported in the literature compared to Type C sites. Chemical consumption is in general higher at Salton Sea than in Upper Rhine Graben. For example, 2.4 kg of CaO/kg Li_2_CO_3_ at Salton Sea and 0.1 kg of CaO/kg Li_2_CO_3_ at Upper Rhine Graben are required. The main reason for the higher demand of chemicals is the increased impurity content in Salton Sea’s brine compared to that from Upper Rhine Graben.^27^

### Comparison with the Literature

We compare our results with the literature LCI of Li_2_CO_3_ production from brines (Table B.5). The compilation of scattered literature data reveals discrepancies arising from (1) data aggregation, (2) LCI completeness, and (3) selected technical parameters.

#### Data Aggregation

Several studies rely on aggregated company data or simplified process details, limiting transparency and prohibiting any in-depth analysis on these differences. For example, our modeled heat demand for Atacama (18 MJ/kg Li_2_CO_3_) is higher than the ecoinvent v3.9.1 value (2.9 MJ/kg Li_2_CO_3_), which is based on outdated company data without detailing specific processes to achieve battery-grade quality.^24^ Kelly et al.^24^ relied on company data for the same site including fewer chemicals—only Na_2_CO_3_ is explicitly mentioned, and the value reported is similar to ours. Freshwater is estimated to be 0.024 m^3^/kg of Li_2_CO_3_, which is half of our modeled value. An explanation might be that their freshwater demand includes enhanced recirculation strategies than our conservative assumptions. However, the lacking disclosure of this type of information prohibits any further analysis.

Mousavinezhad et al.^75^ use aggregated industry data from the Clayton Valley site (type C) and report an energy demand (sum of heat and power: 59 kW h/kg Li_2_CO_3_) which is half compared to ours (e.g., range of summed heat and power: 102–188 kW h/kg Li_2_CO_3_). The lack of transparency on process-related data (i.e., process temperature to heat the raw brine) limits comparison. Our study includes Mg and Ca removal by Na_2_CO_3_, while Mousavinezhad et al.^75^ report acidification as the only required pretreatment process at Clayton Valley, explaining the lower Na_2_CO_3_ demand (1.5 kg/kg Li_2_CO_3_) in their study compared to our range (1.9–38 kg/kg Li_2_CO_3_).

Huang et al.^82^ only assess the DLE process without any pretreatment of the geothermal brine at Salton Sea, leading to a substantial lower resource demand compared to our obtained one (e.g., power use: 1.18 kW h/kg Li_2_CO_3_ compared to 14 kW h/kg Li_2_CO_3_). Additionally, the authors do not report any heat demand which stands in contrast to our results as volume reduction processes, such as the mechanical evaporator, require heat.^83^ In comparison with the results obtained by Schenker et al.^27^ is that the heat demand is reduced from 230 MJ/kg Li_2_CO_3_ to 179 MJ/kg Li_2_CO_3_ in the case of Salton Sea. This results from the enhanced recirculation of process water after Li_2_CO_3_ is precipitated on our model as the heated water can be used to reduce freshwater and energy demand for the adsorption column. In the specific context of water use, recirculation and purification strategies result in a modeled overall water demand (357 kg/kg Li_2_CO_3_) which is comparable with the reported range (200–600 kg/kg Li_2_CO_3_) from companies operating at Salton Sea.^84^ However, information on the processing technology including pre- and post-purification processes from these companies is absent, impeding a further comparison with our study.

#### LCI Completeness

Some of the variance stems from incomplete LCI as either relevant processes or inputs and outputs. For example, Schomberg et al.^25^ report additional LCI for Hombre Muerto, Uyuni, and Silver Peak by using linear adaptions of the LCI^39^ based on the Li concentration of the brine. However, this approach does not take into account any technological differences among these sites, such as the use of quicklime at Silver Peak for Mg removal.^62^ Consequently, our site-specific LCI converge from them.^25^

#### Selected Technical Parameters

Underlying assumptions when using technical parameters to model LCI substantially differ between the studies. Our study has a higher heat demand (465 MJ/kg Li_2_CO_3_) than the one (298 MJ/kg Li_2_CO_3_) by Schenker et al.^22^ due to the increased operating temperature (80 °C) in the DLE column than the one (10 °C) reported by Schenker et al.^22^ The increased temperature in the DLE column is often reported in more recent literature and thus used in our recent study.^33,35,85^

Mas-Fons et al.^26^ used a process simulation tool showing higher energy (power and heat) (11.1 kW h/kg Li_2_CO_3_) than our value (6.2 kW h/kg Li_2_CO_3_). This observation is in accordance with Parvatker and Eckelman^86^ that process calculation has the tendency to underestimate process-specific electricity demand compared to process simulation tools.

Furthermore, the water use by Mousavinezhad et al.^75^ is optimistic, assuming full recycling of water, while our model accounts for freshwater withdrawals in a more conservative manner. More specifically, our model sends 90% of process water back after Li_2_CO_3_ precipitation and discards the rest as liquid waste, which still is a rather optimistic assumption.

### Life Cycle Impacts of Li_2_CO_3_ from Brines

**Figure 3:**
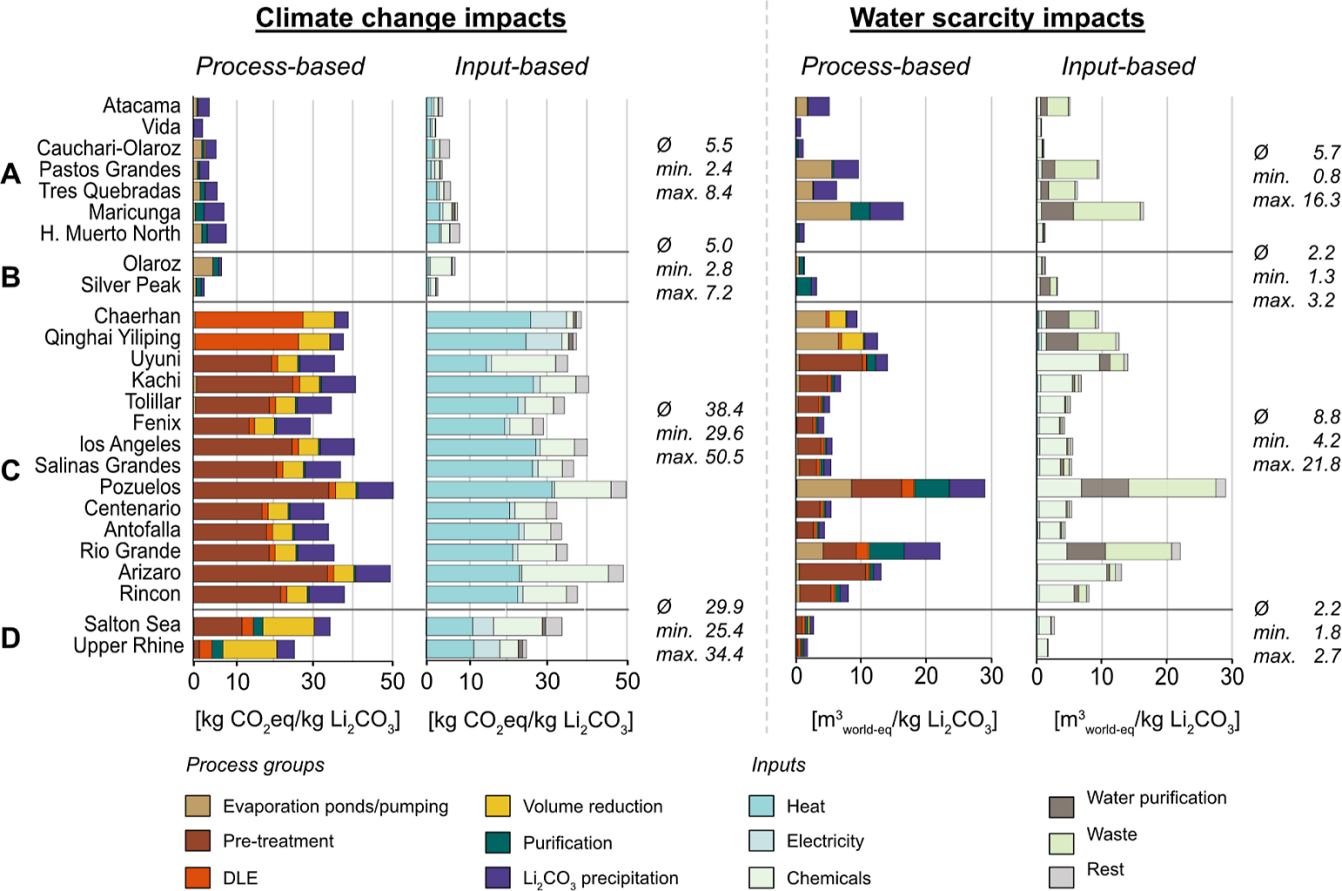
Process- and input-related contributional analysis of climate change and water scarcity impacts of 1 kg of Li_2_CO_3_ at the battery grade of the investigated sites. (A) Conventional chemical-based technology, (B) conventional ion exchanger technology, (C) DLE technology from continental brines, and (D) DLE technology from geothermal brines.

Climate change and water scarcity impacts of 1 kg of Li_2_CO_3_ at the battery grade from 25 sites grouped by technology groups are shown in Figure 3. Additionally, we discuss the site-specific life cycle impacts of the current and future brine-related Li_2_CO_3_ market. The relevance of the energy provision level by using renewable energy on-site and the implications on LIB cell production and the overall raw material sector are discussed.

### Conventional Chemical-Based Technology—Type A

Type A sites have the lowest average climate change impact of 5.5 kg of CO_2_eq/kg Li_2_CO_3_. The range is between 2.4 and 8.4 kg of CO_2_eq/kg Li_2_CO_3_. The impacts mainly originate from chemicals used in evaporation ponds, purification, and Li_2_CO_3_ precipitation. At sites like Cauchari-Olaroz, CaO usage in evaporation ponds is a major contributor due to its heat demand and CO_2_ release during production.^22,87^ For water scarcity impacts, conventional technology sites average 5.7 m^3^ world-eq/kg Li_2_CO_3_, with a range from 0.8 to 16.3 m^3^ world-eq/kg Li_2_CO_3_. These impacts also stem from the chemicals used in evaporation ponds, purification, and Li_2_CO_3_ precipitation. The use of Na_2_CO_3_ amplifies water scarcity impacts due to water losses during chemical production, such as the dehydration of NaHCO_3_ to produce Na_2_CO_3_, which releases H_2_O into the atmosphere.^88^

### Conventional Ion Exchanger Technology—Type B

Type B sites reveal climate change impacts comparable with Type A (Olaroz: 7.2 kg of CO_2_eq/kg Li_2_CO_3_ and Silver Peak: 2.8 kg of CO_2_eq/kg Li_2_CO_3_). Water scarcity impacts are 1.3 m^3^world-eq/kg Li_2_CO_3_ at Olaroz and 3.2 m^3^world-eq/kg Li_2_CO_3_ at Silver Peak. These impacts are mainly due to CaO use in evaporation ponds and ion exchangers. As Silver Peak has a lower impurity concentration than Olaroz (Table 1), Silver Peak uses less CaO than Olaroz to remove impurities, resulting in climate change impacts lower than those of Olaroz. Even though the overall water demand is lower at Silver Peak, it still reveals higher water scarcity impacts than Olaroz due to a characterization factor of 95 m^3^ world-eq/m^3^ at Silver Peak compared to 5 m^3^ world-eq/m^3^ at Olaroz.

### DLE Technology—Type C

Type C sites have higher climate change impacts than the other types, while water scarcity impacts show a more diverse pattern. Li_2_CO_3_ production averages 38 kg of CO_2_ eq/kg Li_2_CO_3_, ranging from 30 to 51 kg of CO_2_ eq/kg Li_2_CO_3_. Within type C, the impacts are determined by the Li concentration in a first order and by the impurity concentration in a second order. Lower Li concentrations generally result in higher climate change impacts, but for the same Li concentration, higher impurity concentrations reveal higher impacts. The main causes for climate change impacts are heat, particularly from natural gas, before the DLE column, and the use of Na_2_CO_3_ and HCl in the pretreatment phase. Chaerhan and Yiliping represent unique cases as they mainly rely on volume reduction processes and do not report any pretreatment phase before the DLE column.^64^ Thus, the climate change impacts are attributed to the DLE process. Water scarcity impacts average 10 m^3^ world-eq/kg Li_2_CO_3_, ranging from 4.2 until 22 m^3^ world-eq/kg Li_2_CO_3_. The most relevant contributors are chemicals used in the pretreatment phase.

### DLE Technology—Type D

Li_2_CO_3_ production from geothermal brines averages 30 kg of CO_2_ eq/kg Li_2_CO_3_ and 2.2 m^3^ world-eq/kg Li_2_CO_3_. Main contributors for climate change are pretreatment and volume reduction processes. Higher impacts are observed for the case of Salton Sea than for Upper Rhine Graben.^27^

### Comparison with the Literature

Climate change impacts of Type A (2.4–8.4 kg CO_2_ eq/kg Li_2_CO_3_) are in the range of the literature (2.1–15.1 kg CO_2_ eq/kg Li_2_CO_3_) (Table B.5). Studies on the Atacama report reveal impacts ranging between 2.1 and 4.9 kg of CO_2_ eq/kg Li_2_CO_3_, while this study reveals climate change impacts of 4.1 kg of CO_2_ eq/kg Li_2_CO_3_. All studies identify Na_2_CO_3_ as the main contributor to these emissions, which is also shown in this study (Figure 3). Notably, Lagos et al.^89^ suggest that the ecoinvent data set underestimates GHG emissions, reporting 1.6 kg CO_2_ eq/kg Na_2_CO_3_ in GREET compared to 0.44 kg CO_2_eq/kg Na_2_CO_3_ in ecoinvent. Using the GREET database, soda ash is the most relevant contributor of climate change impacts and other chemicals become less relevant.^89^ However, required chemicals (HCl and CaO) also rely on generic data leading to an mis-estimation of climate change impacts.^90^

Mousavinezhad et al.^75^ report climate change impacts between 17.3 and 22 kg CO_2_ eq/kg Li_2_CO_3_ at Clayton Valley. These results are lower than the average of the Type C sites (38.4 kg of CO_2_ eq/kg Li_2_CO_3_) due to lower heating demand assumptions. Their energy demand from the industry is toward the lower end of the range reported by Vera et al.,^33^ which, on the other hand, is primarily based on lab-scale data. Our study underlines the importance of the heat demand but also emphasizes the importance of chemicals used in the pretreatment phase. However, the opaque LCI prohibits any detailed evaluation regarding technical differences such as process temperature and chemical use. Schenker et al.^22^ report 31.6 kg of CO_2_ eq/kg Li_2_CO_3_ for Chaerhan, which is lower than our 39 kg of CO_2_ eq/kg Li_2_CO_3_, due to the higher operating temperature of the DLE process in our model.^33^

Regarding Type D sites, the reported value^27^ is 40% lower than 30 kg of CO_2_ eq/kg Li_2_CO_3_ modeled in this study (Figure 3), which is a result of the changes in the heating demand due to enhanced recirculation of the heated process water in our study. Huang et al.^82^ underestimated climate change impacts (2.1 kg of CO_2_ eq/kg Li_2_CO_3_) due to missing pretreatment processes, although they are described in the literature.^15,91^

### Decarbonized Energy Provision

Continental brines are located in regions with a high solar irradiation, making them optimal locations to use solar energy when producing Li_2_CO_3_.^92^ Changing the energy provision to photovoltaic and Cu plate collectors on-site enables a significant reduction potential of climate change impacts (Table B.8). Type A sites show a reduction potential between −20% and −63%. Type B sites have a reduction potential of −49% at the Olaroz peak and −43% at the Silver Peak. The reduction potential is even more pronounced for Type C sites due to their energy-intensive processes. The reduction of climate change impacts varies between −47% at Uyuni and −89% at Chaerhan and Yiliping. The high variability of reduction potentials comes from the use of chemicals (e.g., Na_2_CO_3_, CaO, and sulfuric acid) which mainly contribute to the overall climate impacts and hence are not reduced by on-site measures of alternative energy supply. Furthermore, we tested the reduction potential by implementing a country-specific decarbonized energy provision in the foreground system but did not integrate any decarbonization strategies in the background system. The integration of renewable energies in the mining sector is only one of various levers to decarbonize metals.^93^ Decarbonization strategies in the background could reveal additional reduction potentials. Prospective tools, such as premise,^94^ should be used with caution for this particular endeavor because specific trajectories on future South American electricity mixes are currently missing. Furthermore, prospective assessments on global decarbonization strategies of entire sectors (e.g., chemicals) are needed to capture reduced climate change impacts of any Li chemical production in the future and to highlight any benefits and trade-offs.

### Life Cycle Impacts from a Market Perspective

Using market data from 2023^10^ indicates that the average climate impact of currently producing sites is 15 kg of CO_2_ eq/kg Li_2_CO_3_, while the average climate change impact of late-stage exploration sites is 24 kg of CO_2_ eq/kg Li_2_CO_3_ (+161%) (Figure A4). Regarding early-stage exploration sites, the average of climate change impacts increases up to 37 kg of CO_2_ eq/kg Li_2_CO_3_ (+246%). This strong trend of increasing impacts is less observed for water scarcity impacts. We find that the average of producing sites is 4.0 m^3^ world-eq/kg Li_2_CO_3_, while late-stage and early-stage sites exhibit an average of 6.8 and 9.1 m^3^ world-eq/kg Li_2_CO_3_, respectively.

### LIB Cell Production

The raw material sector is one of the most significant contributors to greenhouse gas emissions of LIB cell production.^95,96^ However, using generic and highly aggregated LCI obscure the range of climate change impacts of LIB cells.^97^ When integrating site-specific LCI of active Li_2_CO_3_ mines into NMC811 batteries, climate change impacts vary between 73 kg CO_2_ eq/kW h and 99 kg CO_2_ eq/kW h of battery capacity, which is in accordance with the 90% confidence interval (59–115 kg CO_2_ eq/kW h) of NMC811 cells.^98^ This stands in contrast to LFP batteries as the relative variance contribution of LFP cells is dominated by Li_2_CO_3_ of 56% in contrast to only 5% of NMC811 cells.^98^ Climate change impacts vary between 54 and 86 kg of CO_2_eq/kWh of LFP battery capacity in our study, while the 90% confidence interval is reported to be between 54 and 69 kg CO_2_ eq/kW h.^98^ Besides Li_2_CO_3_ being a key driver for climate change impacts of LFP cells, our results exceed the confidence interval of LFP cells because our results of currently producing sites (2.8–39 kg of CO_2_ eq/kg Li_2_CO_3_) are higher than the ones obtained in their study (3.1–22 kg of CO_2_ eq/kg Li_2_CO_3_). Additionally, we find that impacts of both battery types increase when using Li from early-stage sites compared to those from currently active mine sites (Table B.9). Early-stage exploration sites require sound assessment at the appropriate time as they can substantially increase the impacts of a battery. The New Battery Regulation by the European Union^99^ defines the reporting of greenhouse gas emissions over the life cycle stages of a battery and aims to reduce the overall carbon footprint of batteries. Our study underlines the need to obtain detailed LCI on the process level^100^ when estimating climate change impacts of batteries in the future.

### Robustness of the Model

**Figure 4:**
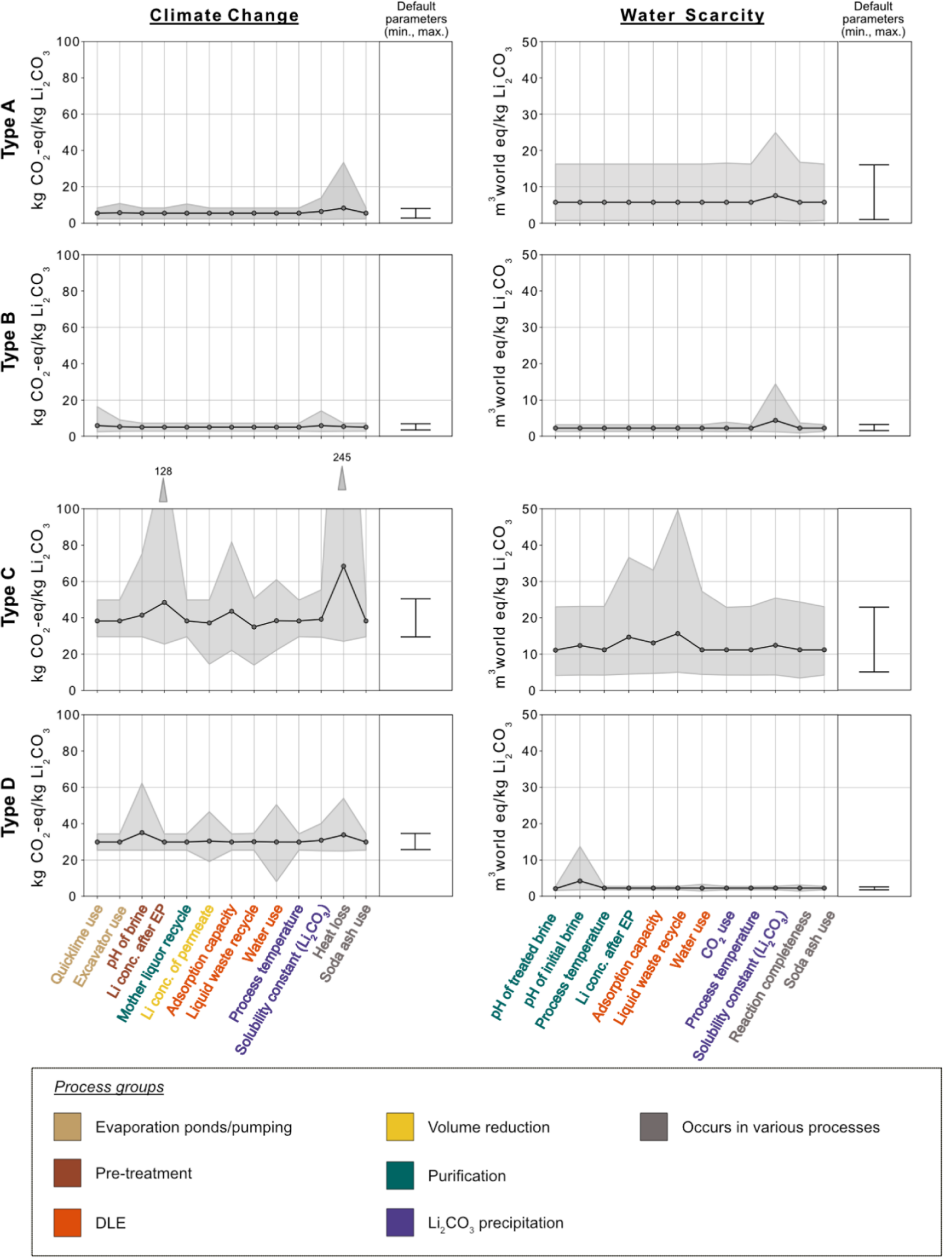
LSA of the defined technology groups. The lines represent the mean value of aggregated sites belonging to this type for the LSA result for each parameter. A threshold of the top 5% of the parameters having the highest standard deviation is applied for this graph. The lower limit of the filled area is the minimum values, and the upper limit is the maximum values of the LSA (Table B.11). The lines on next to the right axis represent the minimum and maximum value without any parameter variation. (A) Conventional chemical-based technology, (B) conventional ion exchanger technology, (C) DLE technology from continental brines, and (D) DLE technology from geothermal brines.

The lack of industrial data requires LCI modeling, which can be in the form of process simulation tools or enhanced process calculations.^86^ However, these modeling approaches come with a high number of parameters, requiring sensitivity analyses. Figure 4 reveals that the sensitivity of parameters considerably varies between the technologies. While type A and B show only small variability in terms of climate change impacts, type C and D show significant sensitivity. For example, heat loss of processes is the most outstanding parameter for type C due to their relatively high heat demand compared to the other types. Variable water scarcity impacts of type A are less affected by the range of the defined parameters but a result of the site-specific conditions (e.g., aridity and brine chemistry).

In addition to the parameter-related sensitivities, the processing sequence of the different sites exhibit uncertainties because the data are obtained from sources with different publication years, detail grades, and system boundaries. For example, Type C sites in this study use similar purification steps since pre- and post-treatment processes are barely site specifically reported.^33^ We use the DLE technology with the highest Technology Readiness Level^33,44^ because other technologies are less well covered in the literature. The model only contains one type of adsorbent, which strongly affects the adsorption capacity, water demand for desorption, and the lifetime of the resin.^33^ Furthermore, the model assumes a constant adsorption capacity, which in reality would decrease over time.^85^ Required pretreatment processes are often not reported, while they are dominating impacts in most cases. The different publication years yield another uncertainty since time-related changes in processes are not included. Besides technological uncertainties, the reported brine chemistry in Table B.2 yields uncertainties as the sampling procedure, location, and time vary between the used sources. Chemical analyses of South American salt lakes^55^ are used and reveal the substantial variability of impacts as a function of brine chemistry within the same site (Figures A5–A7).

### Implications for Li and Other Metals

As the Li demand will increase over the coming years, it is inevitable to ramp up production and open new sites. There is a need to understand the magnitude and the influential site-specific factors of life cycle impacts. Our study shows that the life cycle impacts of Li_2_CO_3_ and their causes vary among the 25 assessed sites. Producing sites have lower life cycle impacts than exploration sites, resulting from a combination of brine chemistry, local conditions, and applied technology.

The model provides detailed insights into site-specific life cycle impacts. This study emphasizes to consider not only Li concentrations but also impurity concentrations, as they influence the required purification processes and thus life cycle impacts. Future Li-related LCA research should focus on the relation between geological constraints and applied processing technology. Using geological data (e.g., from geological surveys) in combination with process simulation tools could reveal more insights into the variability of life cycle impacts of the mining sector.

The increase of life cycle impacts of the assessed market is primarily caused by the widespread implementation of DLE technologies at new sites, which often feature lower Li grades than existing operations. Although DLE technologies have the potential to access previously unreachable Li deposits,^33^ they come with uncertainties due to limited technological data. Existing sites also face these uncertainties when planning to integrate DLE technologies with potentially higher impacts than the technology currently used. Future research should focus on addressing these uncertainties and closing these data gaps.

It is of utmost importance to improve the regionalization of supply chains from a life cycle perspective, especially for water scarcity impacts. As this study relies on generic supply chain data, top-down approaches (e.g., multiregional input–output analysis or trade-related mass flow analysis) should be integrated in future research to improve the localization of environmental hotspots along supply chains.

The future development of the Li market will be shaped by changes in production capacities, electricity mixes, and background supply chains. Technological advancements must be accounted for, especially in such a rapidly developing sector. This includes assessing prospective market development and considering midterm and long-term perspectives, as was shown for cobalt by van der Meide et al.^101^ Additionally, future research should focus on assessing Li extraction from both primary (e.g., clays and pegmatites) and secondary resources. In particular, Li extraction from secondary resources is still in its infancy and requires scientific assessments for its sustainable development.

Beyond Li, the environmental impacts of other metals required for renewable energy technologies need to be thoroughly assessed in the future (e.g., as demonstrated for the case of copper by Adrianto et al.^102^). This necessity is underscored by recent political measures such as the European Union’s Battery Regulation and the Critical Raw Materials Act, which aim to ensure the sustainable and responsible sourcing of essential materials. These regulations mandate stringent environmental standards and transparency in supply chains, highlighting the importance of comprehensive and transparent data from the mining sector.

## Data Availability

Github link to the model to perform a global regionalized life cycle assessment of Li_2_CO_3_ from brines: https://github.com/ecological-systems-design/LiBrineLCAModel.

## Supporting Information Available

The Supporting Information of this manuscript contains the following files The Supporting Information is available free of charge at https://pubs.acs.org/doi/10.1021/acs.est.4c12619.

Overall model structure to perform a regionalized life cycle assessment and required input data, detailed process description and used sources, and further results on brine chemistry and local sensitivity analysis (PDF)
Raw data on used AWARE characterization factors, input data for the model, results on resource demand, and provision of site-specific life cycle inventories (XLSX)
