# Supplementary material for: Current and Future Impacts of Lithium Carbonate from Brines: A Global Regionalized Life Cycle Assessment Model
Source: Environ Sci Technol. 2025 Mar 26;59(13):6543–55. doi: 10.1021/acs.est.4c12619 (PMC11984106; doi:10.1021/acs.est.4c12619)
Supplement: Supplementary file 1 — es4c12619_si_001.pdf [file es4c12619_si_001.pdf]

Appendix A

Current and Future Impacts of Lithium Carbonate from Brines: A Global Regionalized Life Cycle Assessment Model

Vanessa Schenker and Stephan Pfister

Swiss Federal Institute of Technology Zurich, Chair of Ecological Systems Design,

Laura-Hezner-Weg 7, CH-8093 Zurich

Appendix A includes:

- Number of pages: 34
- Number of figures: 7
- Number of tables: 5

Supporting information A contains additional description on the LCI model. Section "LCI modeling" gives an overview of the LCI model and gives more insights in technologies and the defined processes. Details on input data and data gap filling are found in this section as well. More LCA results are documented in section "Life cycle impact assessment" including analyses on brine chemistry (section "Brine chemistry") and local sensitivity analyses (section "Local sensitivity analysis"). Appendix B contains more details on used characterization factors, site-specific LCI, and raw data on LCA results.

List of Figures

A1 *Overview of our presented regionalized life cycle assessment model.* S5

A2 *Overview of our presented life cycle inventory model.* S6

A3 *Life cycle impacts (climate change vs. water scarcity) of Li_2CO_3 production from brines. A: Conventional chemical-based technology, B: Conventional ion exchanger technology from continental brines, C: DLE technology, D: DLE technology from geothermal brines* S30

A4 *Life cycle impacts of Li_2CO_3 production from brines. The x-axis represents the reported/assumed production volume. The diamonds depict the Li-concentration. A: Conventional chemical-based technology, B: Conventional ion exchanger technology from continental brines, C: DLE technology, D: DLE technology from geothermal brines* S31

A5 *Life cycle impacts (climate change and water scarcity) of Li_2CO_3 production from brines (Type A and B). Ata = Atacama, Cau = Cauchari-Olaroz, Ola = Olaroz, Pas = Pastos Grandes* S32

A6 *Life cycle impacts (climate change and water scarcity) of Li_2CO_3 production from brines (Type C excluding Uyuni). Ang = Angeles, Ant = Antofalla, Ari = Arizaro, Cent = Centenario, Fen = Fenix, Poz = Pozuelos, Rin = Rincon, Rio = Rio Grande, SalG = Salinas Grandes* S33

A7 *Life cycle impacts (climate change and water scarcity) of Li_2CO_3 production from brines (Uyu = Uyuni).* S33

List of Tables

1 Material and energy demand per kg of Li_2CO_3 (battery grade). Impurity concentration signed with a $*$ means that there was no information on the impurity concentration and a proxy was used. The column "other" presents the sum of other chemicals used on-site. A: Conventional chemical-based technology, B: Conventional ion exchanger technology, C: DLE technology from continental brines, D: DLE technology from geothermal brines 17

A1 Summary of input parameter for LCI model S7

A2 Overview of references used to assess technology S9

A3 Overview of mapped processes in LCI model S11

A4 Overview of mapped process classes in the life cycle inventory model. S12

A5 Overview of the activity status based on the used database. S28

Life cycle inventory modeling

Overview of LCI modeling approach

Figures A1 and A2 give a general overview of the main steps that the presented model executes to assess the life cycle impacts of 1 kg of Li_2CO_3 at battery grade. Boxes containing < / > refer to specific functions and classes within the model. An excel file (Table A1) is used as an input file being imported into the model. This function checks on any open data gaps which need to be filled by multiple approaches (e.g., using brine chemistry from closest neighbouring site). Then the site is set up by modeling the brine mass going into the processing sequence. The processing sequence comes from the excel file and is matched with the processes mapped in our model. The ProcessManager is a class containing various functions from setting up the variables being transferred within processes but also checking on dependencies between processes. Multiple set-ups can be used to obtain life cycle inventories site-specifically. In total, 30 processes are included in this model and can be expanded in the future. The ProcessManager let the process classes run by the defined sequences. Recirculations of brine or freshwater are taken into account at the end of the modeling. At the same time, the class ResourceCalculator sums up the energy, water, chemical demand and waste production and saves the results in a csv file. Once the processing pathway is modelled, it is transferred into Brightway2. Background database is imported and based on the modeled processing pathway, a site-specific database is created. All in- and outputs are mapped and linked to ecoinvent datasets. For each process, an activity containing the modelled exchanges is generated. Furthermore, some activities require regionalization to account for water scarcity impacts later. These are regionalized by changing the location to the site-specific one. Copies of databases are created to perform further analyses (e.g., energy provision). When the databases are set up, the life cycle impacts are calculated and subsequently, visualized. Local sensitivity analyses can be performed by defining ranges of parameters stored in $operationalandenvironmentalconstants.py$.

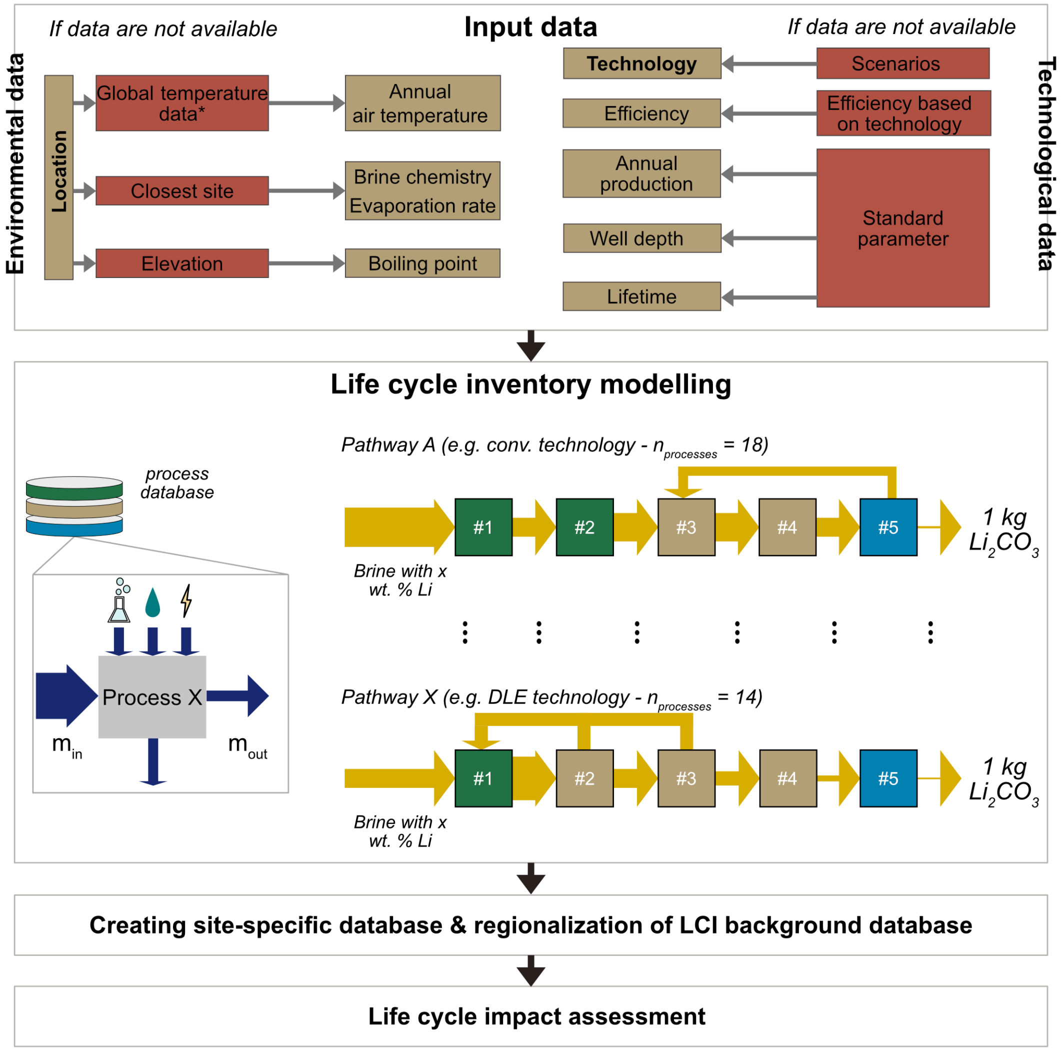

Figure A1: *Overview of our presented regionalized life cycle assessment model.*

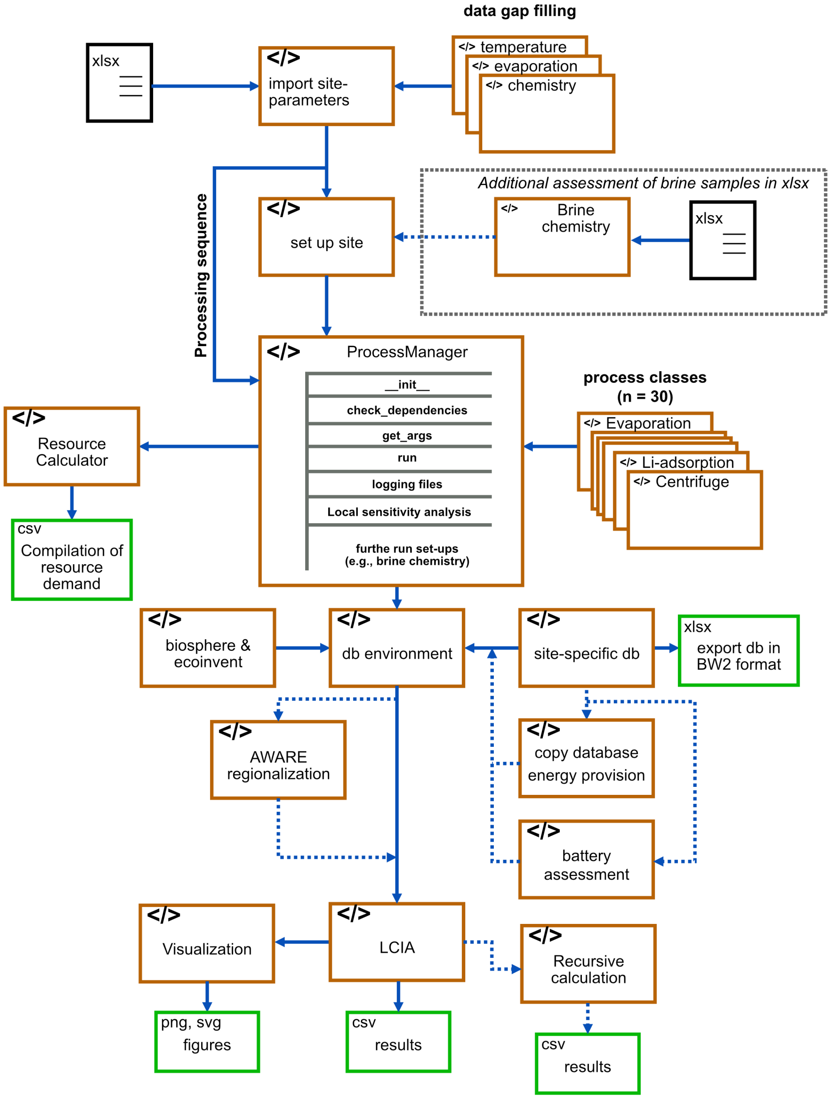

Figure A2: *Overview of our presented life cycle inventory model.*

Input data and data gap filling

Required site-specific input data are found in the supporting information B. An overview of input parameters is given in Table A1. In addition to that, Table A2 reports the used references to assess the technology of each site.

Table A1: Summary of input parameter for LCI model

**Input parameter**	**Explanation**	**If no data is available**	**Standard value in dictionary**
**site name**	Used to create database in BW2	Core parameter	—
**abbreviation**	Used for various application	—	—
**deposit_type**	Options are:<br>– geothermal<br>– salar<br>– oilfield could be added	Core parameter	—
**activity_status**	Activity status by SPGlobal	No adaption is needed;<br>just for visualization	—
**technology_group**	Options are:<br>– salar_conv (A)<br>– salar_IX (B)<br>– salar_DLE (C)<br>– geo_DLE (D)	Core parameter	—
**country**	—	—	—
**elevation**			—
**longitude**	—	Core parameter	—
**latitude**	—	Core parameter	—
**annual_airtemp**	Annual air temperature (average)	Monthly copernicus data are used (2019);<br>Closest data set is used.	—
**evaporation_rate**	Evaporation rate in mm/year	Closest site that reports evaporation rate is used	—
**boilingpoint_process**	Boiling point at elevation	Calculating boiling point based on elevation	—
**density_brine**	Density of Li-bearing brine [g/cm^3]	from standard value dictionary	1.33
**vec_ini**	Chemical composition of Li-bearing brine [wt. %]		—
**ini_Li**			
**ini_Ca**			
**ini_Mg**			
**ini_SO4**			
**ini_B**			
**ini_Si**		Closest site that reports brine chemistry	
**ini_Mn**			

Table A1 continued from previous page

Input parameter	Explanation	If no data is available	Standard value in dictionary
**ini_Fe**			
**ini_Zn**			
**ini_Sr**			
**ini_Ba**			
**ini_H2O**			
**density_enriched_brine**	Density of enriched Li-bearing brine [g/cm^3]	from standard value dictionary	
**vec_end**	Chemical composition of Li-bearing brine [wt. %]; If DLE is used, then the same initial brine chemistry is used to model processing sequence		—
**end_Li**			
**end_Ca**			
**end_Mg**			
**end_SO4**			
**end_B**			
**end_Si**		If required, then closest site is used. If DLE is used, then no proxy is required.	
**end_Mn**			
**end_Fe**			
**end_Zn**			
**end_Sr**			
**end_Ba**			
**end_H2O**			
**production**	Annual production mass [kg]	from standard value dictionary	10000000
**operating_days**	Operating days per year	from standard value dictionary	90% × 365
**lifetime**	Reported lifetime of the mine [year]	from standard value dictionary	30
**brine_vol**	Pumped brine volume [L/s]	Based on production and Li-concentration	—
**freshwater_reported**	Options for freshwater demand reported on-site: – 1: yes, – 0: no	from standard value dictionary	—
**freshwater_vol**	Pumped freshwater volume [L/s]	Proxy is used	—
**Li_efficiency**	Reported overall efficiency of the mine	Based on technology-group	40 %
**number_wells**	Wells pumping the brine to evaporation ponds/processing facility [amount]	from standard value dictionary	10
**well_depth_brine**	Depth of wells [m]	from standard value dictionary	50
**well_depth_freshwater**	Depth of wells [m]	using well depth from brine	—
**distance_to_processing**	Distance to processing facility	from standard value dictionary	0

Table A1 continued from previous page

Input parameter	Explanation	If no data is available	Standard value in dictionary
**quicklime_reported**	Quicklime used in evaporation ponds.<br>Options:<br>– 1: yes,<br>– 0: no	from standard value dictionary	0
**second_Li_enrichment_reported**	Additional information on Li-concentration in evaporation ponds.<br>Options:<br>– 1: yes,<br>– 0: no	from standard value dictionary	0
**second_Li_enrichment**	Li-concentration of enriched brine in evaporation ponds	only used if second_Li_enrichment_reported = 1	—
**diesel_reported**	Diesel demand in evaporation ponds.<br>Options:<br>– 1: yes,<br>– 0: no	from standard value dictionary	0
**diesel_consumption**	Reported diesel demand for annual Li_2CO_3 production	only used if diesel_reported = 1	using proxy from Salar de Atacama
**motherliq_reported**	Mother liquor in process description.<br>Options:<br>– 1: yes,<br>– 0: no	from standard value dictionary	0
**process_sequence**	Required processing sequence used in the model (process_1, process_2, etc.)		

Table A2: Overview of references used to assess technology

Site name	Literature for technology
Salton Sea	Stringfellow and Dobson[15], Schenker et al.[27], Featherstone et al.[42], Warren[43]
Upper Rhine Graben	Schenker et al.[27], Goldberg et al.[44],[56], Vulcan Energie Ressourcen GmbH[57], Castanos-Mollor and Bray[58]
Salar de Atacama	Schenker et al.[22], Stamp et al.[23], Sociedad Mineral Salar de Atacama[59]

Table A2 continued from previous page

**Site name**	**Literature for technology**
Salar de Olaroz	Schenker et al.[22], Orocobre[32]
Salar de Cauchari-Olaroz	Schenker et al.[22], Burga et al.[45]
Salar del Hombre Muerto North	Schenker et al.[22], Goodwin et al.[46]
Tres Quebradas	King and Dworzanowski[47]
Salar del Rincon	Nicolaci et al.[48]
Salar de Arizaro	Rosko[49]
Salar de Rio Grande	Nicolaci et al.[48]
Salar de Tolillar	Millard et al.[50]
Fenix	Nicolaci et al.[48]
Sal de Vida	Gunn et al.[52]
Salar de Pastos Grandes	Reidel et al.[65]
Salar de Antofalla	Nicolaci et al.[48]
Salar de Centenario	Nicolaci et al.[48]
Pozuelos	Nicolaci et al.[48]
Salar de Salinas Grandes	Brooker[66]
Maricunga	Reidel et al.[61],[67]
Salar de Uyuni	Zhang et al.[68]
Silver Peak	USGS[62]
Kachi	Lake Resources[63]
Chaerhan	Schenker et al.[22], Lanke Lithium[64]
Qinghai Yiliping	Xu et al.[74]
Sal de los Angeles	Spanjers[53]

Modeling of processing sequence

Table A3 gives an overview of the mapped processes in the model. The names also refer to the processes listed in Table A4.

Table A3: Overview of mapped processes in LCI model

**Process Name**	**Class name in model and required in input xlsx file**
Evaporation ponds/pumping	evaporation_ponds
DLE evaporation ponds	DLE_evaporation_ponds
Transportation brine	transport_brine
Boron removal by organic solvent	B_removal_organicsolvent
Mg and Ca removal by soda ash	Mg_removal_sodaash
Acidification	acidification
Ca and Mg removal by sodium hydroxide	CaMg_removal_sodiumhydrox
Mg removal by quicklime	Mg_removal_quicklime
Sulfate removal by calcium chloride	sulfate_removal_calciumchloride
Manganese and Zinc removal by quicklime	MnZn_removal_lime
Iron and Silica removal by limestone	SiFeRemovalLimestone
Ion exchanger (high water demand)	ion_exchange_H
Ion exchanger (low water demand)	ion_exchange_L
Mechanical evaporator	triple_evaporator
Reverse osmosis	reverse_osmosis
Nanofiltration	nanofiltration
Li-ion selective adsorption	Li_adsorption
Li_2CO_3 precipitation (TG/low purity)	Liprec_TG
Dissolution	dissolution
Li_2CO_3 precipitation (BG)	Liprec_BG
Washing Li_2CO_3 (BG)	washing_BG
Washing Li_2CO_3 (TG/low purity)	washing_TG
Centrifuge (general)	Centrifuge_general
Centrifuge after Mg removal by soda ash	CentrifugeSoda
Centrifuge Li_2CO_3 (BG)	CentrifugeBG
Centrifuge Li_2CO_3 (TG/low purity)	CentrifugeTG
Centrifuge Washing	CentrifugeWash
CentrifugeQuicklime	CentrifugeQuicklime
Rotary dryer	rotary_dryer

Description of processes

Table A4 gives a general overview of all mapped processes in the model. Furthermore, it gives additional information on sources and explanations for various assumptions made in the model.

Table A4: Overview of mapped process classes in the life cycle inventory model.

**Flows**	**Explanation**
**Process: Evaporation ponds/Pumping**	
General description	The brine is pumped into evaporation ponds in order to increase the Li-concentration to a site-specific threshold[23,24,32].
Inflow/Outflow	Reported pumped brine volume is used to calculate the inflow. Outflow is calculated by using the Li-threshold to calculate the mass of enriched brine going into the next process.
Energy	Energy is required for pumping and removing precipitated salt from the evaporation ponds. Electricity required for pumping is based on Schenker et al. (2022) with the following calculations: Diesel requirements to remove salt are calculated based on the mass of precipitated salt in evaporation ponds. (1) Diesel requirement is given by official report, then this value is used. (2) If no diesel requirement is given, the model uses that as a proxy (precipitated salt/diesel reported at evaporation ponds at Salar de Atacama) to calculate the diesel demand for other sites[59].

Table A4 continued from previous page

Flows	Explanation
Chemicals	Quicklime:<br>(1) *If initial brine chemistry and enriched brine chemistry are reported*: Quicklime demand is calculated by using the difference of Mg and Ca content in the brine (initial brine vs end brine). 20 % additional quicklime is added to account for incomplete chemical reactions[22].<br>(2) *If no brine chemistry is given (except Li)*:<br>Model uses a proxy to estimate quicklime demand. Proxy is based on the quicklime demand of the operation at Salar de Olaroz[32]. Model uses quicklime demand per mass of Mg in the brine pumped into evaporation ponds.<br>Sulfuric acid: Sulfuric acid demand is based on the freshwater demand at the evaporation ponds[26]. The model uses 1 % of the estimated/reported freshwater demand to quantify the sulfuric acid demand[23].
Waste	The amount of salt waste is calculated by the following principles:<br>(1) *Enriched brine chemistry is given*: Based on the difference of the enriched and initial brine chemistry, the amount of waste is calculated in the model.<br>(2) *No enriched brine chemistry is given*: Proxy used from the Salar de Atacama is used to approximate salt precipitated in evaporation ponds. Liquid waste is defined as the water used for pipe washing. The liquid waste is treated by the modeled waste water treatment on site.
Water	Water is used for washing pipes at evaporation ponds and to mix chemical solution, if required. Washing pipes is modeled by:<br>(1) Freshwater demand on-site is reported<br>(2) Freshwater demand is not given: The model uses a proxy from Salar de Atacama[59]. It is based on the reported freshwater requirement per mass of precipitated salt in the evaporation ponds.
Re-circulations	Not applicable
Other	Sites using DLE also contain this process but all resources except energy demand and water demand are set to zero in order to account for pumping activities.
**Process: Transport to processing plant**	
General description	The brine is transported via truck to the processing plant[23].

Table A4 continued from previous page

Flows	Explanation
Inflow/Outflow	The calculated outflow of the evaporation ponds is used as an inflow. No losses are assumed during transportation.
Energy	-
Chemicals	-
Waste	-
Water	-
Re-circulations	-
Other	Diesel consumption is based on the estimated ton-kilometer to the processing plant.
**Process: DLE evaporation ponds/storage ponds**	
General description	The LiCl solution is stored in ponds. No chemicals are added.
Inflow/Outflow	The inflow is the outflow of the previous process. The model only estimates a small volume reduction which is then used as an outflow.
Energy	-
Chemicals	-
Waste	-
Water	-
Re-circulations	-
Other	-
**Process: Boron removal by organic solvent**	
General description	Boron of the enriched brine is removed by using organic solvents. Before the organic solvent is used, the pH of the brine needs some adjustments[16,19,54,60].

Table A4 continued from previous page

Flows	Explanation
Inflow/Outflow	Output of previous process
Energy	The operating temperature is 10 °C based on Wilkomirsky[60]. The model uses the operating temperature and the annual temperature to model the heating demand: $$q = m \cdot \Delta T \cdot C \quad (1)$$ q is the heat [J], m is the mass [kg], Δ T is the temperature difference [K], C is the heat capacity of an aqueous solution<br>If the difference between the operating temperature and annual temperature is negative then no heating is necessary but the model assumes that the brine is cooled down and the residual heat is released into the atmosphere.
Chemicals	Hydrochloric acid: Hydrochloric acid is calculated by considering the buffering capacity of the brine. Hence, the changes in pH[54,60] and the borate and sulfate concentrations are used to estimate the hydrochloric acid demand.<br>Sodium hydroxide: Based on the hydrochloric acid and $Na_2B_4O_7$, the model calculates the sodium hydroxide demand.
Waste	Boron precipitates: The model uses the enriched brine chemistry to calculate the boron precipitates. The following chemical reaction is used: (1) $Na_2B_4O_7 + 2\,HCl + {}_5H_2O\text{-} \longrightarrow {}_4H_3BO_3 + 2\,NaCl$<br>Organic solvent waste: Organic solvent waste is based on the recycling rate of the organic solvent[22,59].
Water	Water is required for:<br>(1) Sodium hydroxide solution<br>(2) Hydrochloric acid solution<br>(3) Stripping water to remove boron from the brine
Re-circulations	Organic solvent is recycled. Recycling rate is based on Sociedad Mineral Salar de Atacama[59].
Other	—
**Process: Mg and Ca removal by soda ash**	
General description	Mg and Ca are removed by adding soda ash to the pulp. Salts ($MgCO_3$ and NaCl) are precipitated. If mother liquor is reported for this site, then the mother liquor is included in this process[60].

Table A4 continued from previous page

**Flows**	**Explanation**
Inflow/Outflow	The inflow is the pulp outflow of the previous process. If mother liquor is reported, then the mother liquor is added to the inflow.
Energy	Operating temperature is set to 60 °C and temperature of incoming flow is set to annual air temperature[60]. If mother liquor is reported, then the temperature of the mother liquor is taken into account. Depending on the temperature of the pulp, either the heating demand is modeled or the heat emissions into the atmosphere are modeled. Heat demand is modelled by the following equation:<br>$$q = m \cdot \Delta T \cdot C \quad (2)$$<br>q is the heat [J], m is the mass [kg], Δ T is the temperature difference [K], C is the heat capacity of an aqueous solution
Chemicals	Soda ash is estimated by enriched brine chemistry (Mg concentration). If no Mg concentration is given, the model uses a the enrichment factor (initial Li/end Li) to estimate the Mg concentration. Based on the following chemical reaction, the soda ash and waste are modelled: $MgCl_2(aq) + Na_2CO_3(aq)\text{-} \longrightarrow MgCO_3(s) + {}_2NaCl(s)$ In addition, 20 % is added on top to account for the incompleteness of the reaction[69].
Waste	Salts ($MgCO_3$ + NaCl) precipitate in this process. They are removed in the subsequent centrifuge so the process does not include any waste flows.
Water	Water is required for:<br>(1) Soda ash solution<br>(2) Quicklime solution
Re-circulations	Not applicable
Other	—
**Process: Acidification**	
General description	Process to adjust pH of incoming pulp. The pH is fixed in this process. The pulp chemistry, assuming the enriched brine chemistry, is taken into account in order to consider the buffering effect of borates and sulfates[15,42].
Inflow/Outflow	Output of previous process
Energy	—

**Flows**	**Explanation**
Chemicals	Hydrochloric acid is modeled based on the changes in pH are reported by Featherstone et al.[42]. Furthermore, the model uses the borate and sulfate content of the brine to estimate the additional hydrochloric acid demand accounting for the buffering capacity.
Waste	—
Water	Water is required for hydrochloric acid.
Re-circulations	Not applicable
Other	
**Process: Mg and Ca removal by sodium hydroxide**	
General description	Process to remove Mg, Ba, Sr, and Ba. Only reported for geothermal brines[15,42,57].
Inflow/Outflow	Output of previous process
Energy	—
Chemicals	Sodium hydroxide is modeled by using a fraction of the brine chemistry entering the processing facility. The left over is set to 0.02 % which is based on Featherstone et al.[42]. Chemical demand and resulting amount of precipitates is calculated by the following chemical reactions:<br>(1) $Mg^{2+} + 2\,NaOH \longrightarrow 2\,Na^{+} + Mg(OH)_2$<br>(2) $Ba^{2+} + 2\,NaOH \longrightarrow 2\,Na^{+} + Ba(OH)_2$<br>(3) $Sr^{2+} + 2\,NaOH \longrightarrow 2\,Na^{+} + Sr(OH)_2$<br>(4) $Ca^{2+}Na_2CO_3 \longrightarrow {}_2Na^{+} + CaCO_3$
Waste	Mg-, Ba-, Sr-hydroxides and calcite are precipitated.
Water	Water is required for a sodium hydroxide solution.
Re-circulations	Not applicable
Other	—
**Process: Mg removal by quicklime**	
General description	Process to remove Mg from the pulp by using quicklime[54].
Inflow/Outflow	Output of previous process

Table A4 continued from previous page

Flows	Explanation
Energy	—
Chemicals	Mg-concentration proxy is used to calculate quicklime demand. The model calculates the chemical demand based on the following chemical reactions:<br>(1) $MgCl_2(aq) + Ca(OH)(aq)- \longrightarrow Mg(OH)_2(s) + CaCl_2(aq)$<br>(2) $MgSO_4(aq) + Ca(OH)(aq)- \longrightarrow Mg(OH)_2(s) + CaSO_4(s)$<br>(3) $Na_2SO_4(aq) + Ca(OH)(aq)- \longrightarrow {}_2NaOH(aq) + CaSO_4(s)$
Waste	Mg- and Ca-precipitates are produced but not removed in this process, but in the following centrifuge.
Water	Water is required for a quicklime solution.
Re-circulations	Not applicable
Other	—
**Process: Sulfate removal by calcium chloride**	
General description	Process to remove sulfates from the pulp by calcium chloride[70].
Inflow/Outflow	Output of previous process
Energy	No information on operating temperature. Model does not take into account any additional heating.
Chemicals	Model uses the enriched brine chemistry to model calcium chloride demand. The model uses the approach by Schenker et al.[22]. 30 % of the SO_4 concentration of the brine is used to model the calcium chloride demand.
Waste	Sulfates are precipitated and removed in this process.
Water	Water is required for a calcium chloride solution.
Re-circulations	Not applicable
Other	Only reported for Salar de Cauchari-Olaroz
**Process: Manganese and Zinc removal by quicklime**	
General description	Process to remove Mn- and Zn-hydroxides from the brine[15,42,57].

Table A4 continued from previous page

Flows	Explanation
Inflow/Outflow	Output of previous process
Energy	No information on operating temperature. Model does not take into account any additional heating.
Chemicals	Model uses the enriched brine chemistry to model quicklime demand based on the following chemical reactions:<br>(1) $Mn^{2+} + Ca(OH)_2 \longrightarrow Mn(OH)_2 + Ca^{2+}$<br>(2) $Zn^{2+} + Ca(OH)_2 \longrightarrow Zn(OH)_2 + Ca^{2+}$<br>In order to account for the incompleteness of the chemical reactions, the model uses a safety factor (+ 20 %).
Waste	Fe- and Mn-hydroxides precipitate and are removed in this process by sedimentation.
Water	Water is required for a quicklime solution.
Re-circulations	Not applicable
Other	Only reported for geothermal brines
**Process: Iron and Silica removal by limestone**	
General description	Process to remove Fe and Si from the brine. Limestone is added to force precipitation of hydroxides and oxides[15,42,57].
Inflow/Outflow	Output of previous process
Energy	No information on operating temperature. Model does not take into account any additional heating as the process is specifically mentioned in the context of already heated geothermal brines.
Chemicals	Model uses the enriched brine chemistry to model limestone demand based on the following chemical reactions:<br>(1) $2\,CaCO_3 + 2\,Fe^{2+} + 3\,H_2O + (1/2)\,O_2 \longrightarrow$<br>$2\,Fe(OH)_3 + 2\,CO_2 + 2\,Ca^{2+}$<br>(2) $3\,CaCO_3 + 3\,H_4SiO_4 + 2\,Fe(OH)_3 \longrightarrow$<br>$Ca_3Fe_2Si_3O_{12} + 3\,CO_2 + 9\,H_2O$<br>In order to account for the incompleteness of the chemical reactions, the model uses a safety factor (+ 20 %).
Waste	Fe- and Si-precipitates are removed from the pulp by sedimentation.
Water	—
Re-circulations	—

Table A4 continued from previous page

Flows	Explanation
Other	Only reported for geothermal brines
**Process: Ion exchanger**	
General description	Ion exchanger to remove Mg, B, and Ca from the incoming pulp[31,32,71].
Inflow/Outflow	Output of previous process
Energy	Electricity demand is calculated by using literature data per mass going through ion exchanger[39].
Chemicals	Hydrochloric acid and sodium hydroxide are used to re-generate ion exchangers.
Waste	Na and Cl emissions based on literature datasets are included[39].
Water	Water is required for the re-generation of the ion exchangers.
Re-circulations	Not applicable
Other	The model contains two types of ion exchangers based on different resource demands. The main reason for this is the water hardness. The model assumes that if brine is treated the resource demand of the ion exchanger is higher than if process water (as in the case of geothermal brine related operations) is treated by ion exchangers. Ion exchanger (L) has a general lower resource demand than ion exchanger (H)[22].
**Process: Mechanical evaporator**	
General description	Process to reduce the mass of the pulp by evaporating the solvent through thermal and mechanical processes. The output is a more concentrated solution. Freshwater is produced as a co-product and can be used for other processes[83].
Inflow/Outflow	The outflow is defined based on a fixed enrichment factor based on given Li-concentrations[42]. If mother liquor is reported, then the outflow is calculated based on the inclusion of mother liquor and not following the enrichment factor[22].
Energy	Electricity and heat demand per input are based on Al-Karaghouli and Kazmerski[83]
Chemicals	—
Waste	—

Table A4 continued from previous page

Flows	Explanation
Water	—
Re-circulations	Freshwater is obtained in this process. The freshwater is sent back to processes with high water demand. Depending on the processing sequence, freshwater is sent back to DLE and/or ion exchangers that require freshwater for re-generation or as a carrier solution.
Other	—
**Process: Reverse osmosis**	
General description	Reverse osmosis is used to reduce the mass of pulp and enrich the residual pulp in terms of Li[42]. In this process, freshwater is produced as a co-product and re-used in other water-intensive processes.
Inflow/Outflow	The outflow is defined based on a fixed enrichment factor based on given Li-concentrations[42].
Energy	Electricity demand per mass treated in the reverse osmosis unit is taken from Li et al.[69].
Chemicals	—
Waste	—
Water	—
Re-circulations	Freshwater is obtained in this process. The freshwater is sent back to processes with high water demand.
Other	—
**Process: Nanofiltration**	
General description	Nanofiltration is used to reduce the mass of pulp and enrich the residual pulp in terms of Li[64]. In this process, freshwater is produced as a co-product and re-used in other water-intensive processes.
Inflow/Outflow	No information on any enrichment factor. Hence, the model assumes a factor of 20.
Energy	Electricity demand per mass treated in the nanofiltration unit is taken from Li et al.[69].
Chemicals	—
Waste	—

Table A4 continued from previous page

Flows	Explanation
Water	—
Re-circulations	Freshwater is obtained in this process. The freshwater is sent back to processes with high water demand.
Other	—
**Process: Li-adsorption**	
General description	The Li-adsorption process includes the adsorption, and desorption phase. Li from the brine is adsorbed by the Al-hydroxide resin. Freshwater is used in the desorption phase.
Inflow/Outflow	Brine mass is defined as the inflow. Based on the resin capacity, the freshwater mass is defined used in the desorption phase. We use an average resin capacity given by Vera et al.[33] for continental brines and a resin capacity given by Isupov et al.[72] for geothermal brines.
Energy	Energy demand is calculated by the brine temperature entering the adsorption column. Operating temperature is set to 80 °C due to the increased efficiency[33]. Desorption temperature is set to 40 °C[64]. Depending on the freshwater temperature (i.e., coming from mechanical evaporator, reverse osmosis, nanofiltration), additional heating is required. In the case, freshwater is re-circulated from the aforementioned processes, the heating demand is potentially reduced due to the elevated temperature of the freshwater. The model takes that into account by (1) reducing the freshwater demand and (2) adjusting the energy demand for the direct lithium extraction process. The model uses the operating temperature and the annual temperature to model the heating demand: $$q = m \cdot \Delta T \cdot C \quad (3)$$ q is the heat [J], m is the mass [kg], Δ T is the temperature difference [K], C is the heat capacity of an aqueous solution
Chemicals	The amount of required resin is calculated by the annual lithium carbonate production and adsorption capacity given by literature. We include a loss rate based on DuPont[73] which takes into account the multiple re-use of the resin.
Waste	The amount of resin based on the loss rate is used to determine the resin waste generated in this process.

Flows	Explanation
Water	Based on the resin capacity, the freshwater mass is defined used in the desorption phase. We use an average resin capacity given by Vera et al.[33] for continental brines and a resin capacity given by Isupov et al.[72] for geothermal brines. Freshwater demand is substantially reduced when processes, such as reverse osmosis, mechanical evaporator, and/or nanofiltration are included in the processing sequence. In addition to that, water from the centrifuge after the Li_2CO_3 (TG) precipitation is also sent back to DLE or ion exchanger section to reduce freshwater demand.
Re-circulations	—
Other	—
**Process: Li_2CO_3 precipitation (TG/low purity)**	
General description	Soda ash is added to precipitate Li_2CO_3[19,31,54,60]. Sites using this process at the end of their processing sequence produce technical grade Li_2CO_3[24]. Sites using ion exchanger technology use this process at the beginning and hence, produce Li_2CO_3 at low purity grade[71].
Inflow/Outflow	Output of previous process
Energy	Process temperature is set to 83 °C[16,19,60]. Energy is calculated by using process temperature and depending on the processing sequence, either annual air temperature (ion exchanger technology) or temperature of previous process (conventional technology, DLE). The model uses the operating temperature and the annual temperature to model the heating demand: $$q = m \cdot \Delta T \cdot C \quad (4)$$ q is the heat [J], m is the mass [kg], Δ T is the temperature difference [K], C is the heat capacity of an aqueous solution
Chemicals	Soda ash demand is calculated by using the following chemical reactions[60]:<br>(1) $_2LiCl(aq) + Na_2CO_3\text{-} \longrightarrow Li_2CO_3(s) + {_2}NaCl(s)$<br>30 % surplus is added to account for losses and incomplete chemical reaction.
Waste	NaCl is produced but not discarded in this process but in the following due to the centrifuge.

Table A4 continued from previous page

Flows	Explanation
Water	Water is used for soda ash solution.
Re-circulations	Not applicable
Other	—
**Process: Dissolution**	
General description	To increase purity of Li_2CO_3 up to battery grade, Li_2CO_3 (TG/low purity) is dissolved in water at low temperature and CO_2 is used to improve the dissolution of Li_2CO_3[31,54,60].
Inflow/Outflow	Inflow is defined as outflow of previous process. Outflow is defined based on the solubility of Li_2CO_3 in water. Stated solubility in[60] is at 0.052 kg/kg H_2O.
Energy	Operating temperature is at 10 °C[60]. No required heating or cooling is modeled.
Chemicals	CO_2 is used to improve the dissolution. Ehren and Alem[31] stated a range of excess CO_2 of up to 10 folds. The model assumes that the majority of CO_2 can be captured due to the release in the subsequent process.
Waste	—
Water	Water is modeled based on the solubility of Li_2CO_3 in water. Stated solubility in Wilkomirsky[60] is at 0.052 kg/kg H_2O at 10 °C.
Re-circulations	Not applicable
Other	—
**Process: Li_2CO_3 precipitation (BG)**	
General description	Process is required to precipitate Li_2CO_3 at battery grade.
Inflow/Outflow	Inflow is defined as the outflow from the previous process. Outflow is defined based on the inflow and releases of water and CO_2[19].

Table A4 continued from previous page

Flows	Explanation
Energy	Operating temperature is the site-specific boiling point[54]. Energy is modeled based on the solution temperature and operating temperature. The solution temperature varies depending on the processing sequence (e.g., ion exchanger technology – previous process is ion exchanger at 70 °C)<br>The model uses the operating temperature and the annual temperature to model the heating demand:<br>$$q = m \cdot \Delta T \cdot C \quad (5)$$<br>q is the heat [J], m is the mass [kg], Δ T is the temperature difference [K], C is the heat capacity of an aqueous solution
Chemicals	—
Waste	Heat waste is produced in this process. Heat waste is modeled based on the release of H_2O and CO_2 and their respective heat content.
Water	—
Re-circulations	CO_2 is fully re-circulated to dissolution process
Other	—
**Process: Washing**	
General description	Process required to wash Li_2CO_3 at technical or battery grade[31,42,54,57,60].
Inflow/Outflow	Inflow is defined as the outflow from previous process. Outflow is defined as the functional unit including additional water.
Energy	Operating temperature is boiling point. Required water is heated up to operating temperature.<br>The model uses the operating temperature and the annual temperature to model the heating demand:<br>$$q = m \cdot \Delta T \cdot C \quad (6)$$<br>q is the heat [J], m is the mass [kg], Δ T is the temperature difference [K], C is the heat capacity of an aqueous solution
Chemicals	—
Waste	—

Table A4 continued from previous page

Flows	Explanation
Water	Water is defined as double the mass of the functional unit to wash Li_2CO_3.
Re-circulations	Not applicable
Other	—
**Process: Centrifuge (general and purification)**	
General description	Class of various centrifuges used after processes (e.g., washing of Li_2CO_3, Mg removal by soda ash). The common characteristic of modeled centrifuges is that depending on their use, they produce solid, liquid, re-used waste. The amount of the produced waste is defined in each process separately.
Inflow/Outflow	Inflow is defined as the outflow from previous process. Outflow is defined on a factor used in the centrifuge. For example, after washing of Li_2CO_3 the residual Li_2CO_3 contains 50 water and this then sent to the rotary dryer.
Energy	Electricity per treated mass is calculated by using literature values based on Schenker et al.[22].
Chemicals	—
Waste	3 types of waste exist:<br>(1) solid – all used centrifuges before Li_2CO_3 is precipitated produce solid waste.<br>(2) liquid – all used centrifuges after Li_2CO_3 is precipitated produce liquid waste.<br>(3) re-used (DLE) – special case for sites using DLE. Due to the intensive use of freshwater when desorbing Li, the liquid waste is sent back to DLE or ion exchangers to reduce water demand.
Water	—
Re-circulations	explained in waste
Other	—
**Process: Rotary dryer**	
General description	This process is required to dry Li_2CO_3 at battery grade[31,42,54,57,60].

Table A4 continued from previous page

**Flows**	**Explanation**
Inflow/Outflow	Inflow is defined based on the previous outflow. Outflow is the functional unit.
Energy	Heat and electricity demand per treated mass are defined based on ecoinvent (2022).
Chemicals	—
Waste	50 % of the required heat is modeled to be heat waste in this process.
Water	—
Re-circulations	—
Other	—

Site-specific databases

All sites-specific databases that can be directly imported into Brightway2 are documented at the end of the supporting information B as excel files. As a background database, ecoinvent v3.9.1 is used but can be updated in future, if needed. The exported databases do not contain any regionalized activities for water scarcity impacts to facilitate the import of databases if other life cycle impacts are assessed.

Life cycle impact assessment

In addition to the results presented in the main manuscript, we present some more results. Data used for these figures can be found in the appendix B.

Life cycle impacts

Figure A3 presents life cycle impacts of Li_2CO_3 production in comparison with the reported Li-concentration.

Market perspective Table A5 gives an overview the activity status used in this paper. The grouping is mainly based on S&P Global[10] and used for the discussion on the market perspective in the main paper.

Table A5: Overview of the activity status based on the used database.

**Group**	**Activity Status**
1 - Mine stage	– Production<br>– Operating<br>– Satellite<br>– Expansion<br>– Limited production<br>– Residual production

Group	Activity Status
2 - Exploration - Late stage	– Reserves Development<br>– Feasibility<br>– Feasibility complete<br>– Construction started<br>– Construction planned<br>– Pre-production
3 - Exploration - Early stage	– Grassroots<br>– Exploration<br>– Target Outline<br>– Commissioning<br>– Prefeas/Scoping<br>– Advanced exploration<br>– Feasibility Started

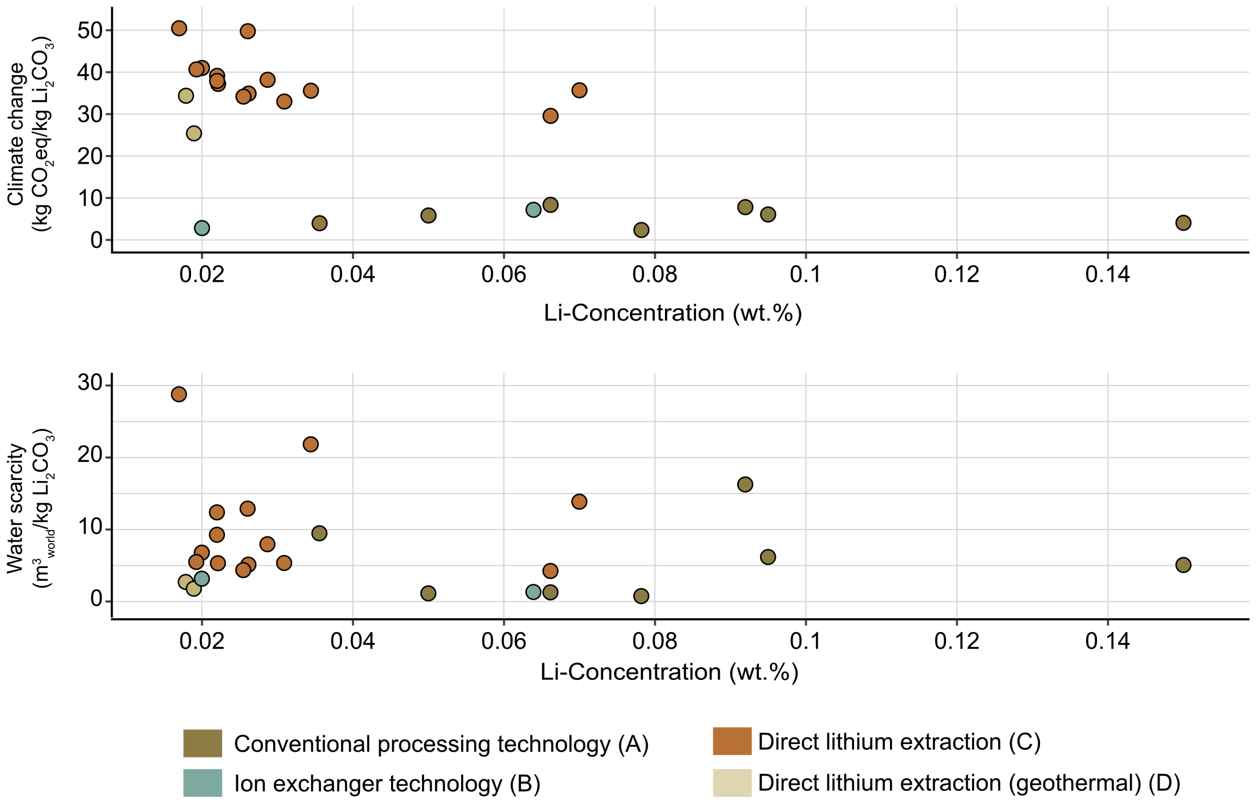

Figure A3: *Life cycle impacts (climate change vs. water scarcity) of* Li_2CO_3 *production from brines. A: Conventional chemical-based technology, B: Conventional ion exchanger technology from continental brines, C: DLE technology, D: DLE technology from geothermal brines*

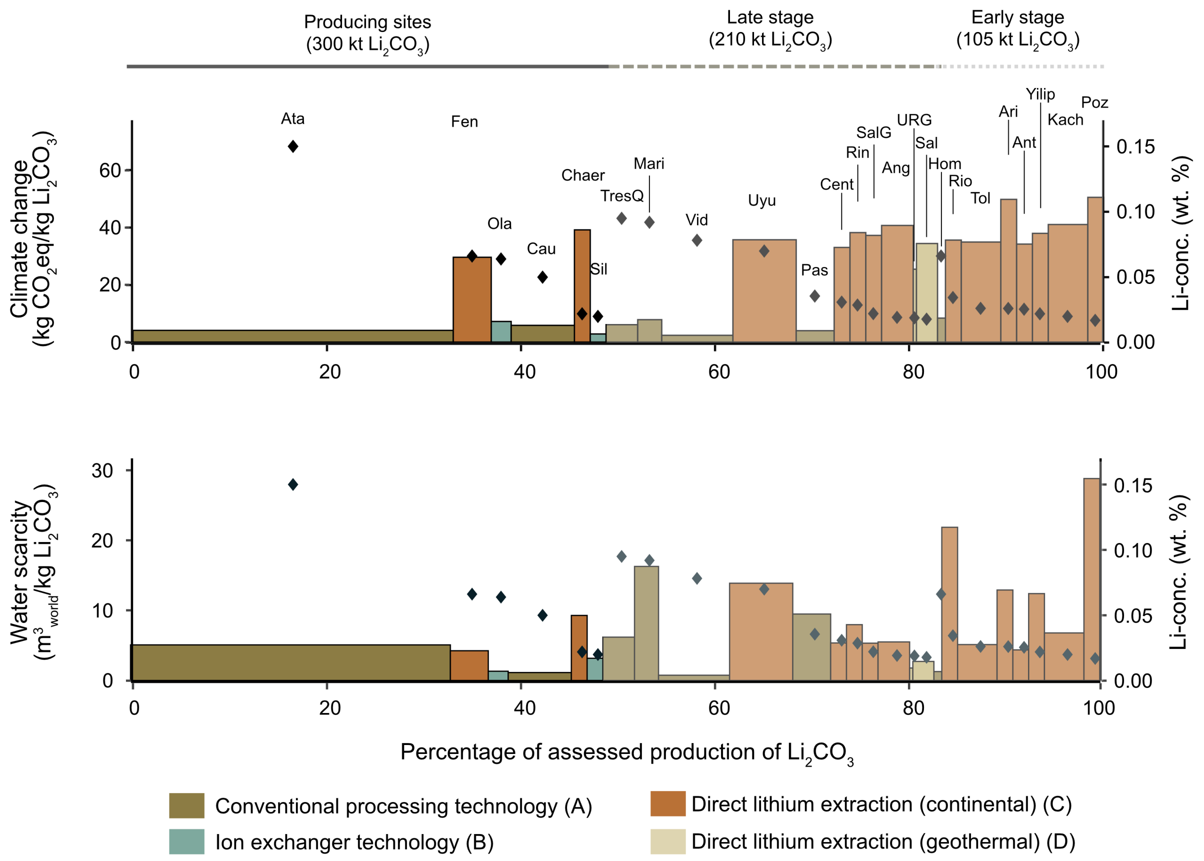

Figure A4: *Life cycle impacts of Li_2CO_3 production from brines. The x-axis represents the reported/assumed production volume. The diamonds depict the Li-concentration. A: Conventional chemical-based technology, B: Conventional ion exchanger technology from continental brines, C: DLE technology, D: DLE technology from geothermal brines*

Figure A4 presents life cycle impacts with the reported/assumed production masses. Used data can be found in Table B.7 of appendix B.

Brine chemistry

Life cycle impacts of Li_2CO_3 production of South American sites are assessed with varying brine chemistry. We used the reported brine chemistry in Steinmetz and Salvi [55]. No economic threshold regarding Li-grade is applied since this assessment is performed to showcase the robustness of the model. The chemical analyses are collected at different times and locations within a single salar which needs to be taken into account. Especially, brine chemistries with a low Li-concentration should be treated with cautions as these brine are likely not economically feasible to extract Li. Raw data can be found in appendix B. Figures A5 to A7 show the variability of life cycle impacts due to the used brine chemistry. Figure A5 showcases sites that belong to technology group A & B, while Figures A6 and A7 showcase sites of technology group C. Since the majority of chemical analyses is based on the Salar de Uyuni, a separate figure showcases Salar de Uyuni.

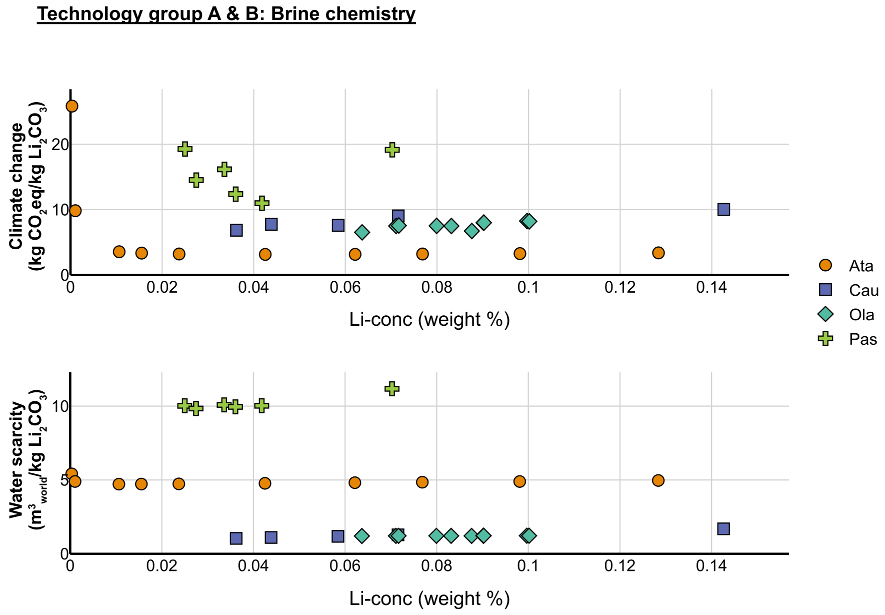

Figure A5: *Life cycle impacts (climate change and water scarcity) of Li_2CO_3 production from brines (Type A and B). Ata = Atacama, Cau = Cauchari-Olaroz, Ola = Olaroz, Pas = Pastos Grandes*

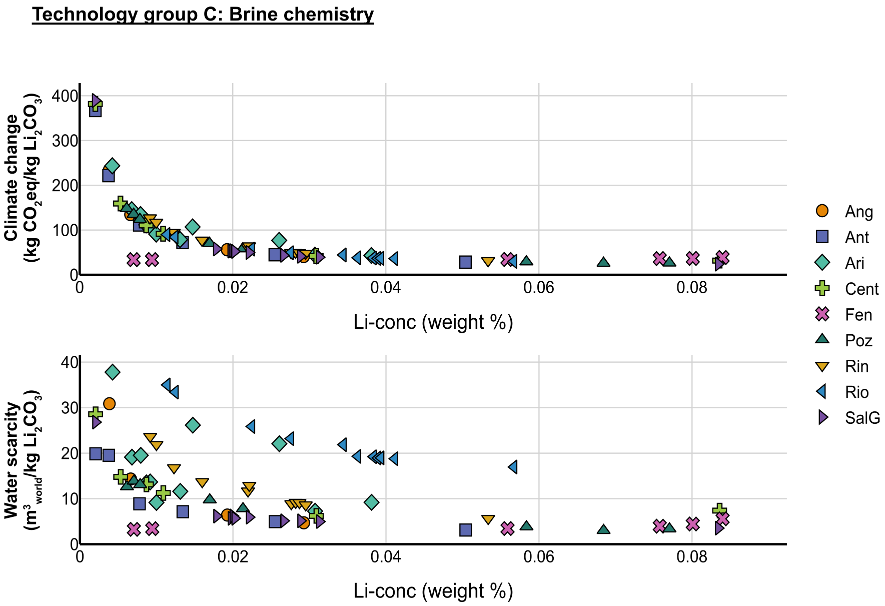

Figure A6: *Life cycle impacts (climate change and water scarcity) of Li_2CO_3 production from brines (Type C excluding Uyuni). Ang = Angeles, Ant = Antofalla, Ari = Arizaro, Cent = Centenario, Fen = Fenix, Poz = Pozuelos, Rin = Rincon, Rio = Rio Grande, SalG = Salinas Grandes*

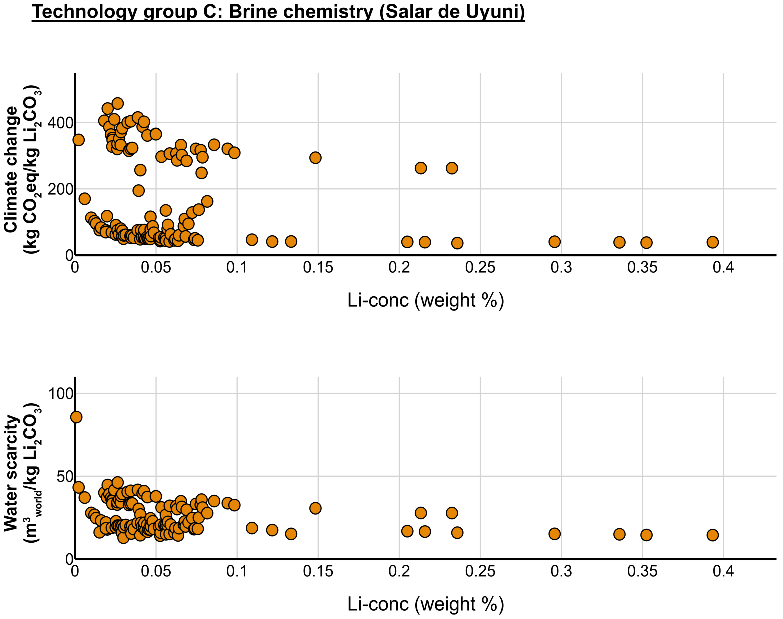

Figure A7: *Life cycle impacts (climate change and water scarcity) of Li_2CO_3 production from brines (Uyu = Uyuni).*

Local sensitivity analysis

In order to evaluate the chosen parameters in our model, a local sensitivity analysis is performed. We defined a range for all parameters in the model and systematically changed them. We developed the following rules to change the parameters in a hierarchical manner.

1. Patent-derived parameters: Parameters obtained from patents are either varied by the reported range in the patent (e.g., process temperature for Li_2CO_3 (TG) precipitation in Wilkomirsky[60]). If no range is reported in the patent, then the parameter is varied between ± 40 %, ± 60 %, and ± 90 %.

2. Parameters derived from scientific literature and/or technical reports: Parameters obtained from scientific literature and/or technical reports often do not report any ranges. If ranges, such as experimental studies of adsorption capacity are collected, as in the case of Vera et al.[33], we use these values. The parameter is varied between ± 40 %, ± 60 %, and ± 90 %. If the parameter is a ratio, then the parameter is varied between 0 - 1 Temperature-related parameters are varied between 0 °C - 100 °C depending on their actual value (e.g., operating temperature for dissolution of Li_2CO_3 at 10 °C - range is defined within the lower temperature field to still allow the dissolution of Li_2CO_3).

3. Parameters obtained from proxies due to lack of literature: The parameter is varied between ± 20 %, ± 50 %, ± 100 %, ± 200 %, and ± 300 %. If the parameter is a ratio, then the parameter is varied between 0 - 1.

For each iteration, we quantified climate change and water scarcity impacts of 1 kg of Li_2CO_3 at battery grade. Table B.10 presents the default values and the according references that are used in the model and the ranges tested in our sensitivity analysis. A local sensitivity analysis was performed for each site. The raw data of the local sensitivity analysis can be found in appendix B.
